# Combination of Orai1 inhibitor CM5480 with specific therapy mitigates pulmonary hypertension and its cardiac dysfunction

**DOI:** 10.1172/jci.insight.191780

**Published:** 2025-11-10

**Authors:** Anaïs Saint-Martin Willer, Grégoire Ruffenach, Bastien Masson, Kristelle El Jekmek, Angèle Boët, Rui Adão, Mathieu Gourmelon, Antoine Beauvais, Jessica Sabourin, Mary Dutheil, Maria-Rosa Ghigna, Laurent Tesson, Séverine Ménoret, Ignacio Anegon, Fabrice Bauer, Vincent de Montpréville, Sudarshan Hebbar, Carmen Brás-Silva, Kenneth Stauderman, Marc Humbert, Olaf Mercier, David Montani, Véronique Capuano, Fabrice Antigny

**Affiliations:** 1 Université Paris-Saclay, INSERM, UMR_S 999, Hypertension Pulmonaire, Physiopathologie and Innovation Thérapeutique (HPPIT), AP-HP, Hôpital Bicêtre, Hôpital Marie Lannelongue (Groupe Hospitalier Paris Saint Joseph), FHU André Cournand, ERN-LUNG, Le Plessis-Robinson, France.; 2 RISE-Health, Department of Surgery and Physiology, Faculty of Medicine, University of Porto, Alameda Prof. Hernâni Monteiro, Portugal.; 3 Department of Pharmacology and Toxicology, School of Medicine, Universidad Complutense de Madrid, Madrid, Spain.; 4 CIBER Enfermedades Respiratorias (Ciberes), Madrid, Spain.; 5 Instituto de Investigación Sanitaria Gregorio Marañón (IiSGM), Madrid, Spain.; 6 Inserm, UMR-S 1180, Signalisation et Physiopathologie Cardiovasculaire, Université Paris-Saclay, Orsay, France.; 7 INSERM, Nantes Université, CHU Nantes, Center for Research in Transplantation and Translational Immunology, UMR 1064, Nantes, France.; 8 Nantes Université, CHU Nantes, INSERM, CNRS, SFR Santé, Inserm UMS 016 CNRS UMS 3556, Nantes, France.; 9 Cardiology Department, Bicêtre University Hospital, Le Kremlin-Bicêtre, France.; 10 Department of Pathology, Groupe Hospitalier-Marie Lannelongue, Le Plessis-Robinson, France.; 11 CalciMedica Inc., La Jolla, California, USA.

**Keywords:** Pulmonology, Vascular biology, Calcium signaling, Cardiovascular disease

## Abstract

Pulmonary arterial hypertension (PAH) is a rare and incurable disease characterized by progressive narrowing of pulmonary arteries (PA), resulting in right ventricular (RV) hypertrophy, RV failure, and eventually death. Orai1 inhibition has emerged as promising therapeutic approach to mitigate PAH. In this study, we investigated the efficacy of a clinically applicable selective Orai1 inhibitor, CM5480, and its effects when combined with standard PAH therapies in a preclinical PAH model. In male and female monocrotaline PAH-rats, CM5480 monotherapy improved hemodynamics, PA, and RV remodeling, as confirmed by RV catheterization, echocardiography, histology, and unbiased RNA-Seq. Standard PAH therapies, ambrisentan or sildenafil, achieved modest improvements in experimental PAH. In contrast, combination therapies with CM5480 yielded significantly greater benefits in reducing PA remodeling and improving cardiac function compared with monotherapies. Furthermore, in vitro experiments showed that Orai1 knockdown reduced pulmonary endothelial cell dysfunction in PAH and that the Orai1 pathway is independent of standard PAH-targeted pathways in PA smooth muscle cells (PASMCs). Finally, we found enhanced Orai1 expression/function in PASMCs and pulmonary vein SMCs from patients with pulmonary veno-occlusive disease. These findings suggest that Orai1 inhibition represents a potentially novel and complementary therapeutic strategy for PAH by acting at pulmonary vascular and RV levels.

## Abstract 2

Our findings demonstrated that Orai1 inhibition represents a novel and complementary therapeutic strategy for pulmonary arterial hypertension by acting at pulmonary vascular and RV levels.

## Introduction

Pulmonary arterial hypertension (PAH) is a rare and devastating disease defined by a mean pulmonary arterial pressure > 20 mmHg, pulmonary capillary wedge pressure ≤ 15 mmHg, and pulmonary vascular resistance (PVR) > 2 Wood units in the absence of chronic lung disease, chronic thromboembolism, or other miscellaneous systemic diseases. PAH results from the progressive narrowing of the distal pulmonary arteries (PA), leading to high PVR, right ventricular (RV) failure (RVF), and death (1, 2). Current PAH therapies target 3 major signaling pathways (prostacyclin, endothelin-1, and nitric oxide) that are all involved in the endothelium-dependent pulmonary arterial tone. Recently, a new antiremodeling therapy (sotatercept), targeting the TGF-β pathway, was demonstrated to improve the hemodynamic and exercise capacity of patients with PAH, opening the way for antiremodeling PAH treatment (3). Despite therapeutic improvements, these therapies do not cure PAH, and lung transplantation remains the ultimate treatment for eligible patients before they reach RVF. RV function is a primary determinant of prognosis in PAH, but it remains understudied and is not targeted by the current specific therapies (4). It is, therefore, essential to develop new therapeutic strategies that target both the RVF and the pulmonary vascular disease.

The pathobiology of pulmonary vascular remodeling in PAH is multifactorial, including PA smooth muscle cells (PASMCs), pulmonary endothelial cells (PECs), and RV cardiomyocytes (1, 5). We recently demonstrated that the pathogenesis of PAH and RVF is mainly due to intracellular calcium (Ca^2+^) signaling mishandling, and we provided proof that the Orai1 Ca^2+^ channel contributes to pulmonary vascular and RV remodeling and could be a therapeutic candidate. Indeed, we demonstrated the efficacy of research-grade Orai1 inhibitors (only used for research) to reduce the development of PAH in experimental PAH models by acting on pulmonary and cardiac levels (6, 7). Unfortunately, the Orai1 blockers used in these studies are unsuitable for clinical use due to a lack of selectivity, short in vivo half-life, or toxicological concerns.

Based on our recent work (6–8), we aim to further validate our proof of concept for Orai1 as a therapeutic target in PAH in combination with current PAH treatments, and with an interest in sex differences. In collaboration with CalciMedica Inc., we tested the Orai1-selective inhibitor, CM5480, which shares properties similar to those of zegocractin (CM4620), a structurally distinct compound currently in clinical development. Building on our previous preclinical work, we aim to assess the efficacy of CM5480 as monotherapy or dual therapy in combination with standard-of-care of PAH, ambrisentan, or sildenafil in the preclinical model of PAH induced in rats by monocrotaline (MCT) exposure.

## Results

### Preventive selective inhibition of Orai1 with CM5480 curbs the development of experimental PAH.

To determine whether Orai1 contributes to the development of PAH induced by MCT exposure, we first assessed the consequences of in vivo pharmacological inhibition of Orai1 by CM5480 by a preventive approach. We administered CM5480 daily at 20 mg/kg by oral gavage from week 1 to week 3 to MCT-PAH rats (Supplemental Table 3; supplemental material available online with this article; https://doi.org/10.1172/jci.insight.191780DS1). We found significant improvement in RV systolic pressure (RVSP), PVR, and RV hypertrophy as assessed by the Fulton index and the morphometric ratio (RV/tibia length) in MCT rats treated with CM5480 compared with MCT+Vehicle rats (Supplemental Table 3).

### Curative inhibition of Orai1 with the selective inhibitor CM5480 lessens the severity of experimental PAH.

**Figure 1:**
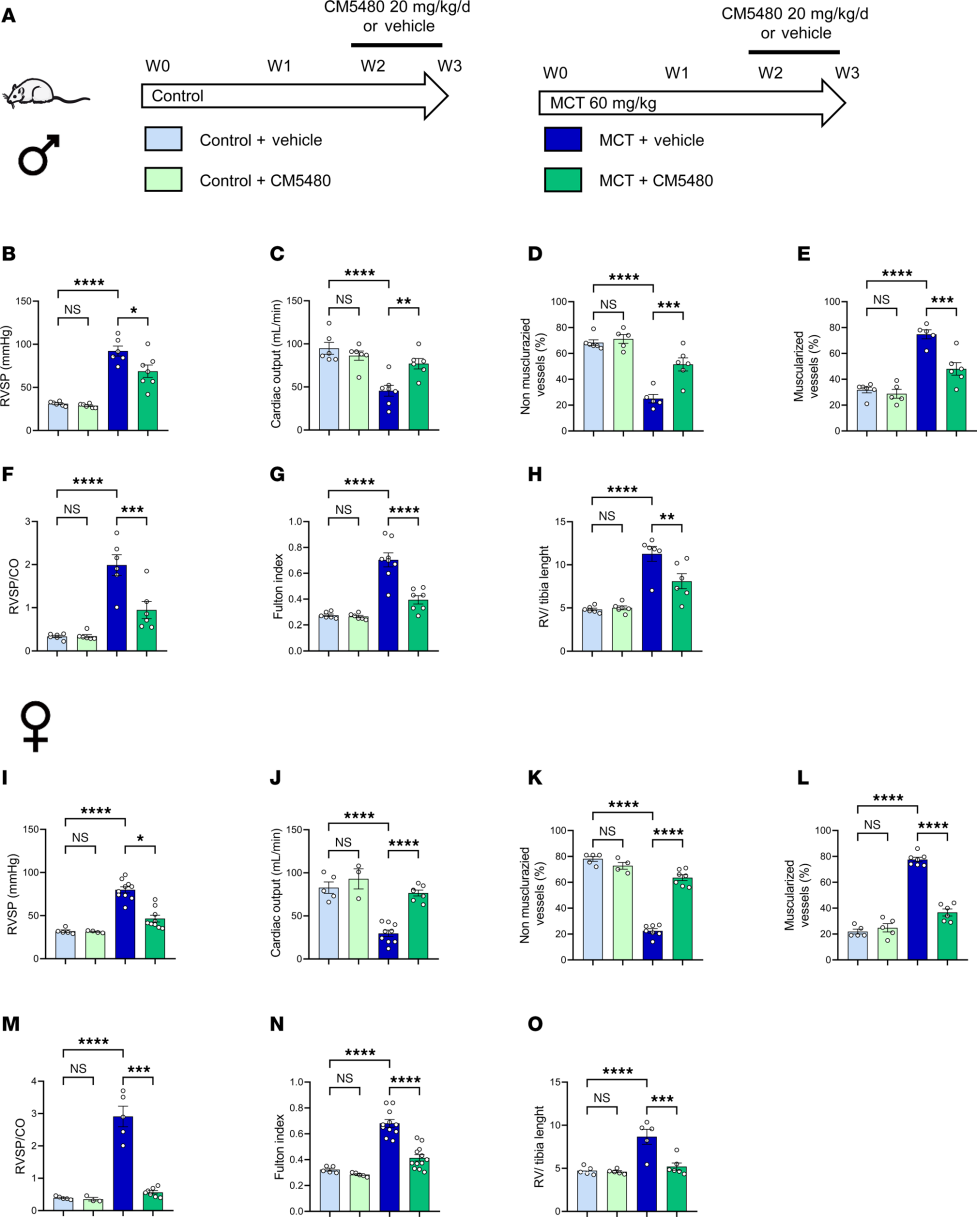
CM5480 monotherapy reduces the severity of PAH induced by MCT exposure in male and female rats considering lung parameters. (**A**) In vivo experimental design. Control and MCT rats (male and female) were treated with vehicle or CM5480 (20 mg/kg/day) by oral gavage for 7 days between week 2 and week 3. (**B**–**H**) Parameters for male rats: RV systolic pressure (RVSP) in mmHg; (*n* = 6 for Control+Vehicle, Control+CM5480, MCT+Vehicle, *n* = 7 for MCT+CM5480), cardiac output (CO) in mL/min (*n* = 6 for Control+Vehicle, Control+CM5480 *n* = 7 for MCT+Vehicle, *n* = 6 for MCT+CM5480), nonmuscularized vessels (*n* = 6 for Control+Vehicle, *n* = 5 for Control+CM5480 and MCT+Vehicle, *n* = 6 for MCT+CM5480), muscularized vessels (*n* = 6 for Control+Vehicle, *n* = 5 for control+CM5480 and MCT+Vehicle, *n* = 6 for MCT+CM5480), RVSP/CO (*n* = 6 for all groups), Fulton index [RV/(LV+septum)] (n=6 for Control+Vehicle, and control+CM5480, *n* = 8 for MCT+Vehicle, *n* = 7 for MCT+CM5480), RV/tibia length (*n* = 6 for all groups) of male control and MCT-rats treated with vehicle or CM5480. (**I**–**O**) Parameters for female rats: RVSP in mmHg; (*n* = 5 for Control+Vehicle, *n* = 4 for control+CM5480, *n* = 10 for MCT+Vehicle, *n* = 9 for MCT+CM5480), CO in mL/min (*n* = 5 for Control+Vehicle, *n* = 3 for Control+CM5480 *n* = 9 for MCT+vehicle, *n* = 6 for MCT+CM5480), nonmuscularized vessels (*n* = 5 for Control+Vehicle, *n* = 4 for control+CM5480, *n* = 7 for MCT+Vehicle and MCT+CM5480), muscularized vessels (*n* = 5 for Control+Vehicle, *n* = 5 for Control+CM5480, *n* = 7 for MCT+Vehicle, *n* = 6 for MCT+CM5480), RVSP/CO (*n* = 5 for Control+Vehicle, *n* = 3 for control+CM5480, *n* = 5 for MCT+Vehicle, *n* = 7 for MCT+CM5480), Fulton index (*n* = 5 for Control+Vehicle and Control+CM5480, *n* = 11 for MCT+Vehicle, *n* = 12 for MCT+CM5480), RV/tibia length (*n* = 5 for Control+Vehicle, Control+CM5480 and MCT+Vehicle, *n* = 6 for MCT+CM5480). Mean ± SEM. One-way ANOVA, Tukey post hoc. *P < 0.05, **P < 0.01, ***P < 0.001, ****P < 0.0001.

In order to determine whether Orai1 inhibition with CM5480 monotherapy could be considered as a new therapeutic avenue to reduce the development of MCT-PAH, we administered CM5480 in vivo from week 2 to week 3 to control and MCT-PAH male and female rats when PAH was established (Figure 1A). In control animals, we found that CM5480 treatment in males had no hemodynamic or morphological effect (Figure 1, B–H, and Supplemental Figure 1). In MCT-PAH male rats, CM5480 therapy reduced RVSP (Figure 1B) and normalized cardiac output (CO) to control values (Figure 1C). Moreover, CM5480 therapy reduced pulmonary neomuscularization, as indicated by the increased nonmuscularized vessels and decreased muscularized vessels in MCT+CM5480 rats versus MCT+Vehicle rats (Figure 1, D and E). Associated with these results, PVR was strongly reduced by CM5480 therapy in PAH rats (Figure 1F). RV hypertrophy was also strongly reduced by CM5480 therapy (Figure 1, G and H).

One notable feature of PAH is its predominance in women (9). Therefore, we assessed the consequences of CM5480 therapy in control and PAH female rats. As in males, CM5480 treatment in control female animals had no hemodynamic and morphological effect (Figure 1 and Supplemental Figure 2). CM5480 therapy in female PAH reduced elevated RVSP (Figure 1I), improved CO (Figure 1J), reduced pulmonary vessel neomuscularization (Figure 1, K and L), and reduced PVR (Figure 1M). RV remodeling was also reduced by CM5480 therapy (Figure 1, N and O). These results demonstrate that CM5480 is an effective therapy for male and female rats with experimental PAH.

### CM5480 treatments reduce RV remodeling and dysfunction in established PAH induced by MCT exposure.

**Figure 2:**
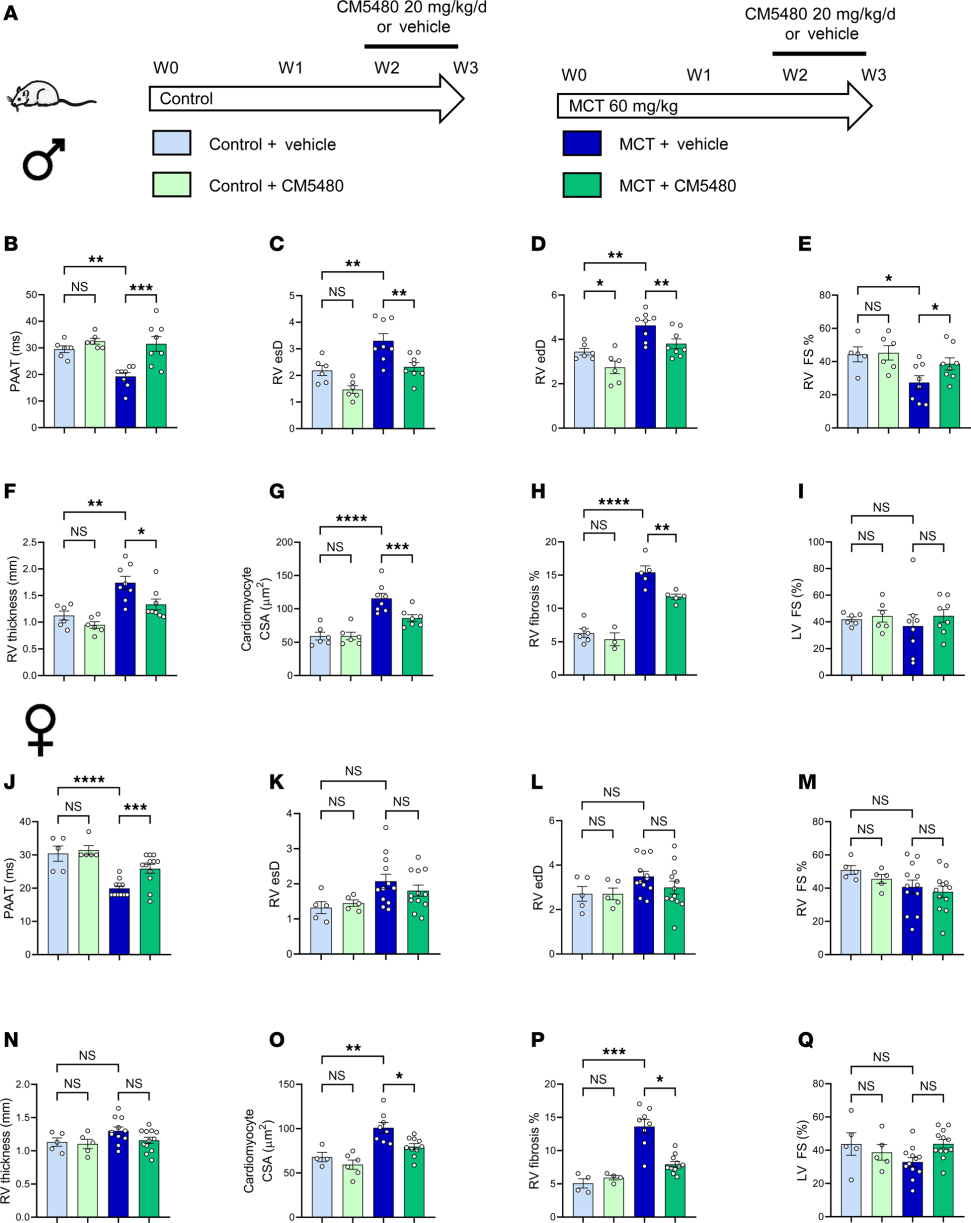
CM5480 monotherapy reduces RV remodeling and dysfunction of MCT-PAH in male and female rats. (**A**) In vivo experimental design. Control and MCT rats (male and female) were treated with vehicle or CM5480 (20 mg/kg/day) between week 2 and week 3. (**B**–**I**) Male rats: PA acceleration time (PAAT) (*n* = 6 for Control group, *n* = 8 for MCT group), RV end-systolic diameter (*n* = 6 for Control group, *n* = 8 for MCT group), RV end-diastolic diameter (RV esD) (*n* = 6 for Control group, *n* = 8 fro MCT group), RV fractional shortening (*n* = 6 for Control group, *n* = 8 for MC group), RV thickness *n* = 6 for control group, *n* = 8 for MCT group), quantification of cardiomyocytes cross-section area (CSA, *n* = 50 cardiomyocytes from 6 rats for control group, 8 rats for MCT+Vehicle and 7 rats for MCT+CM5480). (**H**) Quantification of RV fibrosis by Sirius red staining (*n* = 6 for Control+Vehicle, *n* = 3 for Control+CM5480, *n* = 5 for MCT+Vehicle, *n* = 5 for MCT+CM5480). (**I**) Left ventricle fractional shortening (LV FS) (*n* = 6 for Control group, *n* = 8 for MC group). (**J**–**Q**) Female rats: PAAT (*n* = 5 for Control group, *n* = 11 for MCT+Vehicle, *n* = 12 for MCT+CM5480), RV end systolic diameter (*n* = 5 for Control group, *n* = 12 for MCT group), RV esD (*n* = 5 for Control group, *n* = 12 for MCT group), RV fractional shortening (*n* = 5 for Control group, *n* = 12 for MCT group, RV thickness (*n* = 5 for Control group, *n* = 11 for MCT+Vehicle, *n* = 12 for MCT+CM5480). (**O**) Quantification of CSA (*n* = 50 cardiomyocytes from 4 rats for Control+Vehicle, 6 for Control+CM5480, 8 rats for MCT+Vehicle, and 10 rats for MCT+CM5480). (**P**) Quantification of RV fibrosis (*n* = 4 for Control group, *n* = 8 for MCT+Vehicle, *n* = 11 for MCT+CM5480). (**Q**) LV FS (*n* = 5 for Control group, *n* = 12 for MCT group). Mean ± SEM. One-way ANOVA, Tukey post hoc. *P < 0.05, **P < 0.01, ***P < 0.001, ****P < 0.0001.

In the curative in vivo protocol (Figure 2A), echocardiography analysis revealed that CM5480 in male and female control rats had no effecrt on PA acceleration time (PAAT), RV end-systolic diameter (RVesD), RV end-diastolic diameter (RVedD), RV end-systolic diameter (RVesD), RV fractional shortening (RV FS), RV free wall thickness, RV cardiomyocyte cross-section area (CSA), and RV fibrosis and left ventricle (LV) FS (Figure 2, B–Q).

In PAH male rats, CM5480 therapy restored PAAT, RVedD, RVesD, RV FS, and RV-free wall thickness (Figure 2, B–F). In addition, we observed a decrease in RV CSA and RV fibrosis in CM5480-treated PAH animals (Figure 2, G and H). No significant changes in heart rate, LV parameters, or stroke volume were observed between the vehicle and CM5480 groups (Figure 2I and Supplemental Figure 2).

In PAH females, CM5480 therapy restored PAAT values (Figure 2J). Moreover, despite less RV remodeling in MCT female rats than in MCT male rats (unchanged RV diameter, RV thickness, and RV FS in females) (Figure 2, K–N), we observed a decrease in RV CSA and RV fibrosis in female PAH animals treated by CM5480 (Figure 2, O and P). As in males, no significant change in heart rate, LV parameters, or stroke volume were observed between the vehicle and CM5480 conditions (control and MCT) (Figure 2Q and Supplemental Figure 3). In addition to the effect of CM5480 on pulmonary vasculature, these results demonstrate that CM5480 reduces RV remodeling and dysfunction in male and female rats exposed to MCT.

### Analysis of the consequences of CM5480 treatment on lung transcriptome from control and MCT rats.

**Figure 3:**
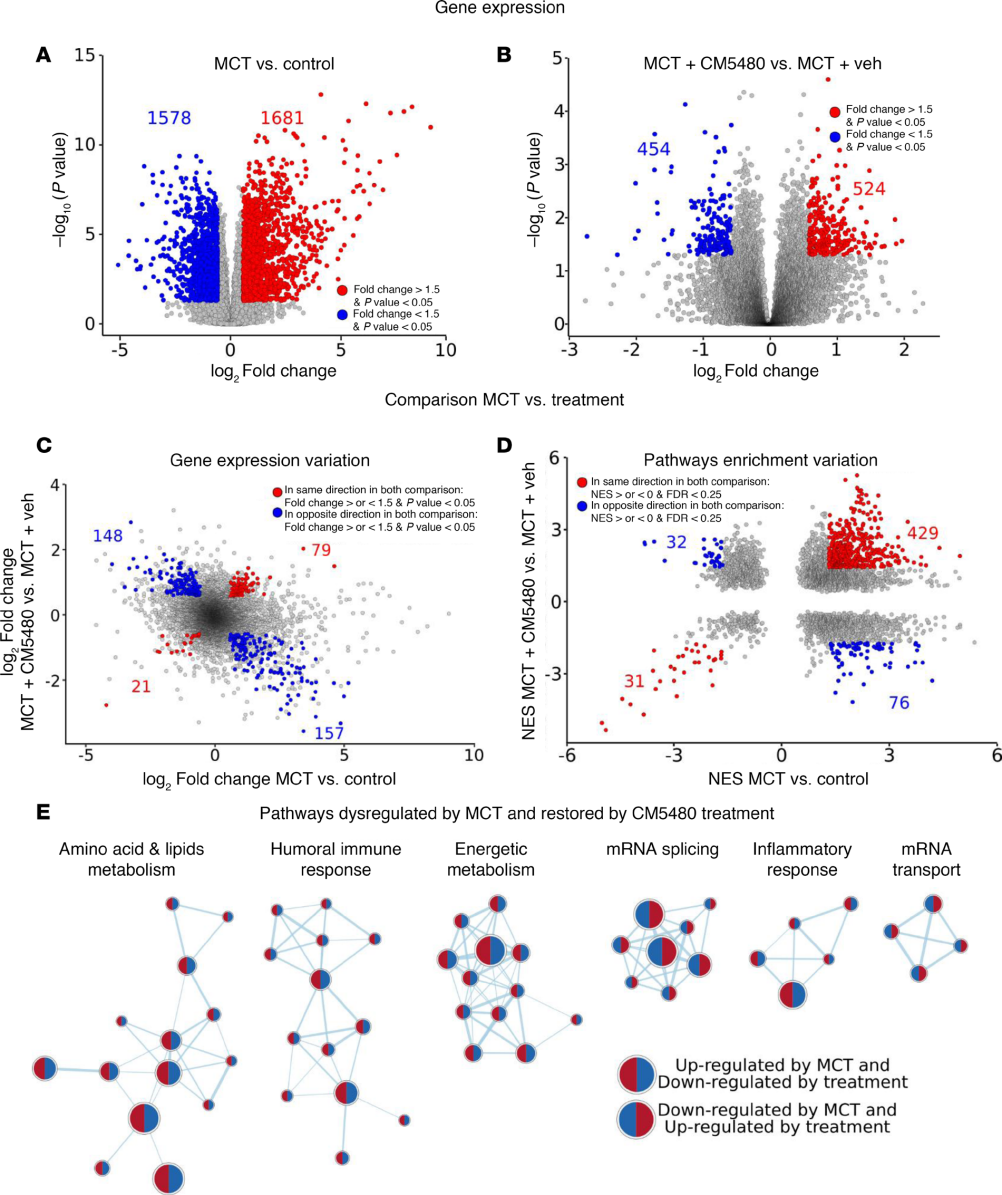
Analysis of the lung RNA-Seq from male rats with CM5480 monotherapy. (**A**) Volcano plot showing gene differentially expressed MCT versus control expressed as –Log_10_ (*P* value) over Log_2_FC. In red, 1,681 upregulated genes (FC > 1.5 and *P* < 0.05). In blue, 1,578 genes were downregulated (FC < 1.5 and *P* < 0.05). (**B**) Volcano plot showing gene differentially expressed MCT+CM5480 versus MCT+Vehicle as –Log_10_ (*P*-value) over Log_2_FC. In red, 524 genes upregulated (FC > 1.5 and *P* < 0.05). In blue, 454 genes were downregulated (FC < 1.5 and *P* < 0.05). (**C**) Gene expression variation for comparison between MCT and treatment expressed as Log_2_FC MCT+CM5480 versus MCT+Vehicle over Log_2_FC MCT versus Control. In red, in the same direction in both comparisons with FC < or > 1.5 and *P* value 0.05. In blue, in the opposite direction in both comparisons with FC < or > 1.5 and *P* value 0.05. For all the RNA-Seq analysis, 5 rats of each group were used. (**D**) Pathway enrichment variation expressed as NES MCT+CM5480 versus MCT+Vehicle over NES MCT versus Control. In red, pathways dysregulated in the same direction in both comparisons: NES > or < 0 and FDR < 0.25. In blue, pathways dysregulated in the opposite direction in both comparisons: NES > or < 0 and FDR < 0.25. (**E**) Pathways dysregulated by MCT and restored by CM5480 treatments. Circles with red on the left and blue on the right for pathways upregulated by MCT and downregulated by CM5480 treatment. Circles with blue on the left and red on the right for pathways downregulated by MCT and upregulated by treatment. Statistical hypothesis testing used Deseq2 R package with Wald test and the Benjamini-Hochberg method for multiple testing.

To determine the molecular consequences of CM5480 therapy in the lung, RNA-Seq analysis was conducted to profile the transcriptome of lung tissues from 5 Control+Vehicle, 5 Control+CM5480, 5 MCT+Vehicle, and 5 MCT+CM5480 male rats. CM5480 minimally altered gene expression in lungs from control rats, supporting the safety of CM5480 in healthy rats (Supplemental Figure 4A). To understand the effect of CM5480 treatment on lung transcriptomic profiles, we first evaluated the putative modification of lung transcriptome in MCT versus control and MCT+CM5480 versus MCT+Vehicle (Figure 3, A and B, and Supplemental Table 4). Then, we assessed the capacity of CM5480 treatment to reverse the altered PAH transcriptomic profile (Figure 3, C and D). Genes were considered significantly dysregulated if the absolute fold change was above 1.5 and the *P* value was below 0.05. This analysis demonstrated that the expression of 305 genes was reversed by CM5480 treatment in experimental PAH. In addition, 100 genes saw the dysregulation amplified by CM5480 (Figure 3C).

Next, we identified the biological pathways modified in PAH and reversed by CM5480 therapy. For this, we used the gene set enrichment analysis (GSEA) algorithm (10, 11) on biological processes from gene ontology gene sets (12, 13) that were considered significantly dysregulated for an FDR < 0.25, as described by the authors of the GSEA algorithm. The lung analysis revealed that pathways such as amino acids and lipids metabolism, immune response, energetic metabolism, and mRNA splicing were among the ones restored by treatment (Figure 3, D and E). These data support a strong therapeutic potential of Orai1 inhibition by CM5480 for the treatment of PAH.

### Analysis of the consequences of CM5480 treatment on the RV transcriptome from control and MCT rats.

**Figure 4:**
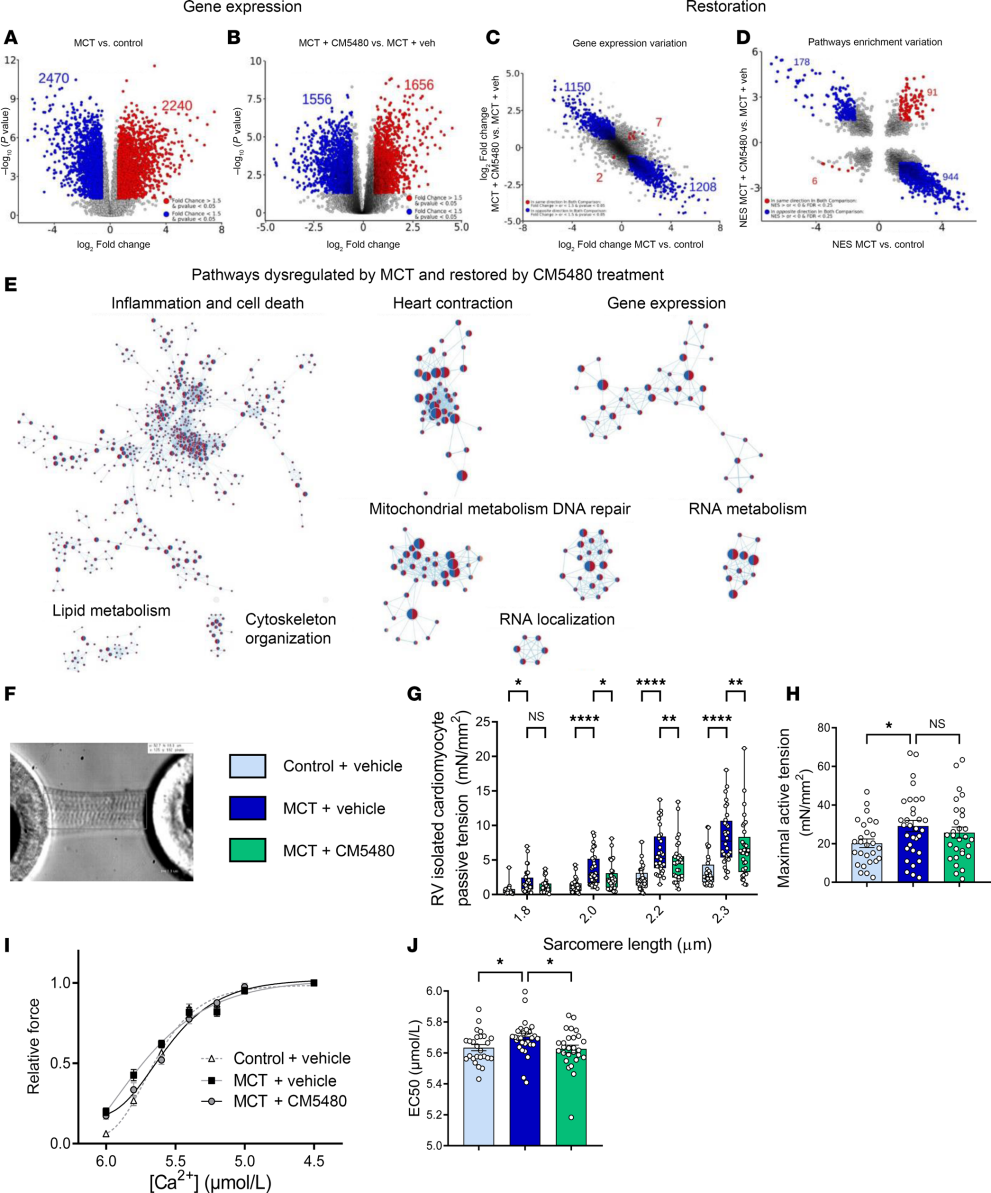
CM5480 monotherapy has a positive effect on RV remodeling in male MCT rats. (**A** and **B**) Volcano plot. In red, upregulated genes (FC > 1.5 and *P* < 0.05). In blue, downregulated genes (FC < 1.5 and *P* < 0.05). (**C**) Gene expression variation between MCT and treatment. In red, genes differentially expressed in the same direction. In blue, genes differentially expression in the opposite direction. (**D**) Pathway enrichment variation expressed as NES MCT+CM5480 versus MCT+Vehicle over NES MCT versus Control. In red, pathways dysregulated in the same direction in both comparisons: NES > or < 0 and FDR < 0.25. In blue, pathways dysregulated in the opposite direction in both comparisons: NES > or < 0 and FDR < 0.25. (**E**) Pathways dysregulated by MCT and restored by CM5480 treatments. Circles with red on the left and blue on the right for pathways upregulated by MCT and downregulated by CM5480 treatment. Circles with blue on the left and red on the right for pathways downregulated by MCT and upregulated by treatment. For all the RNA-Seq analysis, 5 rats of each group were used (same cohort as for lung RNA-Seq). Statistical hypothesis testing used Deseq2 R package with Wald test and the Benjamini-Hochberg method for multiple testing. Experiments performed on frozen RV samples from male rats. (**F**) Image of a single cardiomyocyte attached to a force transducer and a length controller. Scale bar: 1.9 μm. (**G**–**J**) Passive tension in function of sarcomere length (*n* = 32 for each group), maximal active tension (*n* = 28 for Control+Vehicle, *n* = 32 for MCT+Vehicle, *n* = 29 for MCT+CM5480), relative force in function of [Ca^2+^] (*n* same as **H**), EC50 (*n* = 27 for Control+Vehicle, *n* = 32 for MCT+Vehicle and *n* = 29 for MCT+CM5480). (**G**) Mean ± SEM. Two-way ANOVA. (**H** and **J**) 1-way ANOVA. *P < 0.05, **P < 0.01, ****P < 0.0001.

To determine the molecular consequences of CM5480 therapy in the RV, we conducted RNA-Seq to profile the transcriptome of RV tissues from the same cohort of animals as the lung RNA-Seq experiment (Figure 4 and Supplemental Table 5). First, treating control rats with CM5480 induced minimal gene expression alterations, which supports the safety of CM5480 in rats at the RV level (Supplemental Figure 4B). To understand the effect of CM5480 treatment on the RV transcriptome, we evaluated the modifications of the RV transcriptome in MCT versus control and MCT+CM5480 versus MCT+Vehicle (Figure 4, A and B). Then, we assessed the capacity of CM5480 to reverse the altered PAH transcriptomic profile (Figure 4, C and D). To analyze differentially expressed genes, we considered the same fold change and *P* value as lung samples. In RV, the expression of 2,358 genes was reversed by CM5480 therapy in experimental PAH. In addition, only 9 genes exhibited a dysregulation that was amplified by CM5480 treatment (Figure 4C).

We identified biological pathways modified in PAH and reversed by CM5480 treatment. In RV, where the restoration of gene expression was the most striking, we found restoration of pathways involved in inflammation and heart contraction (Figure 4, D and E). Together, these data support a strong therapeutic potential of Orai1 inhibition with CM5480 for treating PAH by restoring most dysregulated pathways involved in RV dysfunction in experimental PAH.

### CM5480 therapy restores the contractile capacity of RV cardiomyocytes from MCT-PAH rats.

In MCT-PAH rats, CM5480 monotherapy reversed abnormalities in cardiac remodeling and function. The active tension of isolated cardiomyocytes was measured using frozen RV samples from Control+Vehicle, MCT+Vehicle, and MCT+CM5480 groups, which we next measured. Single isolated RV cardiomyocytes from MCT+Vehicle animals showed increased passive tension compared with cells isolated from control animals (Figure 4F). In single RV cells isolated from MCT+CM5480 animals, we found that CM5480 treatment partly restored the changes in passive tension (Figure 4G), whereas CM5480 treatment was not able to reduce the increased maximal active tension following the development of PAH (Figure 4H). In addition, RV cardiomyocytes from MCT+Vehicle animals showed increased sensitivity to Ca^2+^ (Figure 4, I and J) compared with Control+Vehicle, which was normalized by CM5480 monotherapy (Figure 4, I and J).

### Histological analyses demonstrated that CM5480 treatment preserves liver, kidney, and spleen structures.

As illustrated in Supplemental Figure 5, the renal architecture is intact, displaying numerous glomeruli with normal morphology. The tubular compartment shows no significant alterations, with no signs of interstitial inflammation or tubular necrosis. Renal vessels are unremarkable and exhibit no observed pathological changes (Supplemental Figure 5A). Moreover, the liver architecture is well preserved in the 4 experimental groups (Control+Vehicle, Control+CM5480, MCT+Vehicle, and MCT+CM5480), with normal alternating portal spaces and centrolobular veins. Portal spaces appear thin, without evidence of inflammatory infiltrates or ductular proliferation. Hepatocytes are arranged in single-cell or double-cell trabeculae within the liver lobule, with no sinusoidal dilation or inflammatory infiltration. There are no signs of steatosis or hepatocellular necrosis. Centrolobular veins remain patent and undilated (Supplemental Figure 5B). In addition, the splenic tissue demonstrates the normal distribution of red and white pulp without notable abnormalities. Megakaryocytes are present within the red pulp. No evidence of pathological lymphoid or inflammatory infiltration was observed (Supplemental Figure 5C). In combination with the data presented in Figures 1–4, these results confirm the safety of CM5480 in control and MCT-PAH rats.

### Orai1 knockdown in PECs from patients with PAH (PAH-hPECs) reduces endothelial cell dysfunction.

**Figure 5:**
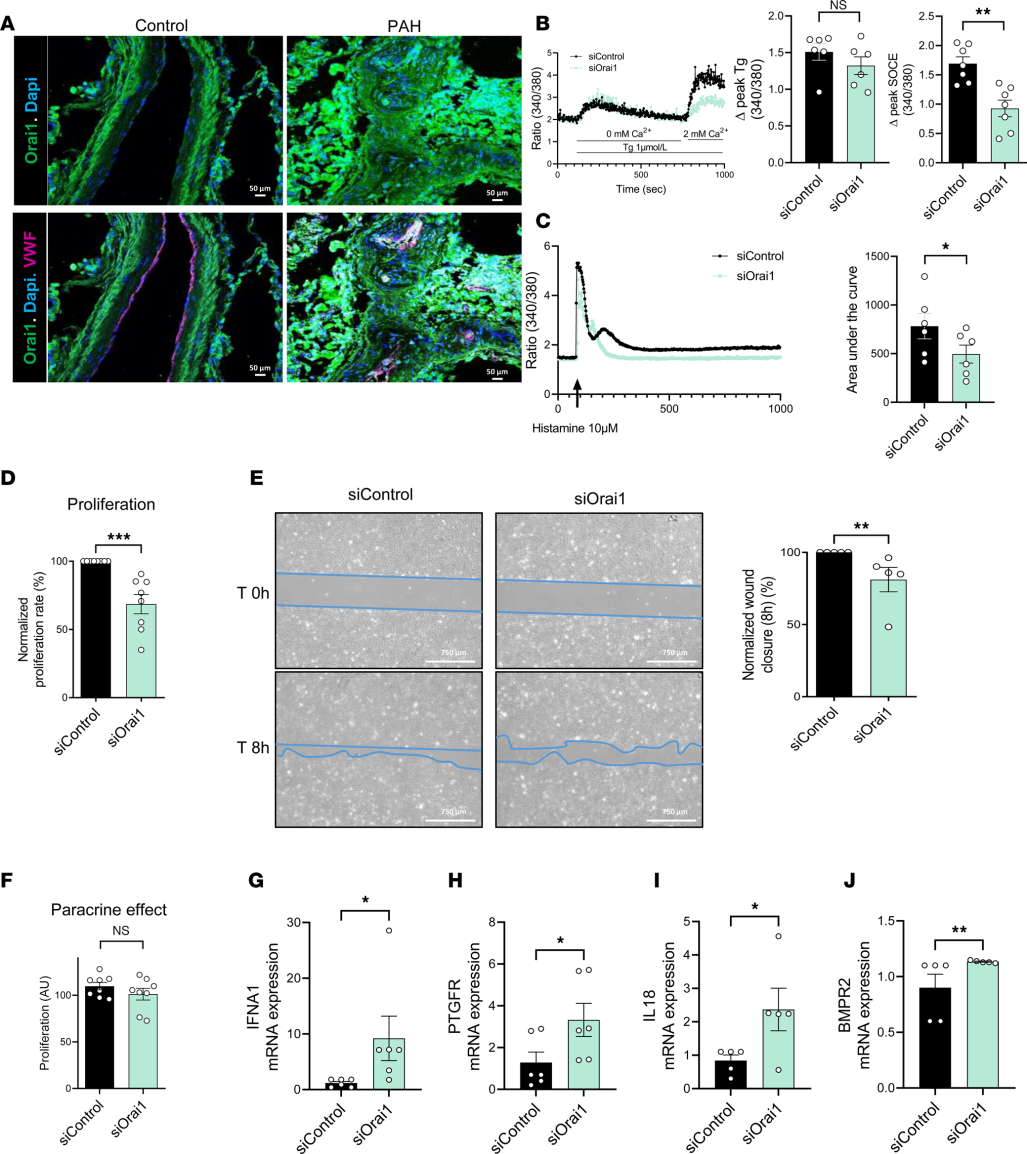
Orai1 knockdown by siRNA on PAH-hPECs reduces pulmonary endothelial dysfunction. (**A**) Localization of Orai1 expression by immunofluorescence staining of paraffin-embedded lung sections from control and patients with PAH. Orai1 is green, vWF is red to localize hPECs, and nuclei are blue (DAPI). Scale bars: 50 μm. (**B**) Consequences of Orai1 knockdown in PAH-hPECs on ER Ca^2+^ release (*n* = 6 patients) and on SOCE amplitude triggered by Thapsigargin (Tg) (*n* = 7 patients). (**C**) Consequences of Orai1 knockdown in PAH-hPECs on the area under the curve after histamine 10 μmol/L stimulation (*n* = 6 patients). (**D**) Consequence of Orai1 knockdown on the proliferation rate (BrdU assay) of PAH-hPECs (*n* = 8 patients). (**E**) Consequences of Orai1 knockdown on PAH-hPECs migration. Wound closure images at time 0 and time 8 hours. Scale bar: 750 μm (*n* = 5 patients). (**F**) Consequence of Orai1 knockdown on the proliferation of control-hPASMCs exposed to PAH-hPECs culture supernatant (*n* = 8 patients). (**G**–**J**) Consequences of Orai1 knockdown in PAH-hPECs on IFNA1 (*n* = 6 patients), PTGFR (*n* = 6 patients), IL-18 (*n* = 5 patients), and BMPR2 (*n* = 5 patients) mRNA expression. Mean ± SEM. Two-tailed Student’s *t* test or Mann-Whitney *U* test. *P < 0.05, **P < 0.01, ***P < 0.001.

Lung RNA-Seq experiments demonstrate the dysregulation of several genes involved in endothelial cell function. The role of Orai1 was investigated in PAH-hPECs using a siRNA strategy to knock down Orai1. Orai1 immunostaining in paraffin-embedded lung sections from control and patients with PAH indicated that Orai1 is expressed in hPECs (Figure 5A), with similar levels between control and PAH. The use of siRNA against Orai1 (siOrai1) reduced store-operated Ca^2+^ entry (SOCE) by 55% in comparison with the siControl condition (Figure 5B) and reduced by 55% the histamine-induced intracellular Ca^2+^ mobilization (Figure 5C). BrdU assay showed that the inhibition of Orai1 reduced PAH-hPEC proliferation by 30% (Figure 5D) as well as PAH-hPEC migration by 20% compared with siControl condition (Figure 5E), without affecting in vitro PAH-hPEC angiogenesis capacity (Supplemental Figure 6A).

We measured the role of Orai1 in the crosstalk between PAH-hPECs and control hPASMCs. The proliferation of hPASMCs was measured by transferring PAH-hPECs medium conditioned with siControl or with siOrai1 to control hPASMCs cultures. Orai1 knockdown has no effect on mitogenic crosstalk in the PAH-hPECs medium (Figure 5F). To better decipher the role of Orai1 in PAH-hPECs function, we performed quantitative PCR (qPCR) assay with 90 endothelial markers. We found that Orai1 knockdown increased mRNA expression of *IFNA1*, prostaglandin F receptor (*PTGFR*), *IL-18*, and bone morphogenetic protein receptor type 2 (*BMPR2*) (Figure 5, G–J). These results indicate that Orai1 inhibition partly improves PAH-hPECs function.

Moreover, no change was observed in the expression of the other 86 endothelial markers, including endothelin1 (*EDN*), endothelin receptor type A and B (*EDNRA, EDNRB*), vascular endothelial growth factor a (*VEGFA*), angiotensin-converting enzyme 2 (*ACE2*), angiotensin 1 converting enzyme (*ACE*), *ICAM1*, and *PECAM1* (Supplemental Figure 6, B–L). We also found that CM5480 reduced SOCE by 85% in PAH-hPECs, without any changes in ER-Ca^2+^ release (Supplemental Figure 7A).

### Role of Orai2 and Orai3 in PAH-hPECs.

Coimmunoﬂuorescence staining with vWF in human lung sections demonstrated that, in addition to Orai1, hPECs from control and patients with PAH expressed Orai2 and Orai3 (Supplemental Figure 7B). In hPECs, we found that Orai2 protein expression was unchanged in PAH-hPECs compared with control-hPECs, while Orai3 protein expression was increased in PAH-hPECs versus control-hPECs (Supplemental Figure 7C). To determine their implication in Ca^2+^ signaling, we used a siRNA strategy to reduce Orai2 or Orai3 expression (Supplemental Figure 7D). As presented in Supplemental Figure 7, E and F, the knockdown of Orai2 or Orai3 had no effect on ER Ca^2+^ release and no effect on SOCE in PAH-hPECs. In addition, in PAH-hPECs treated with siOrai2 or siOrai3, we found that CM5480 strongly reduced SOCE, demonstrating that SOCE in PAH-hPECs is mainly due to Orai1 (Supplemental Figure 7, G and H).

### Role of Orai2 and Orai3 in PAH-hPASMCs.

Coimmunoﬂuorescence staining with α-smooth muscle actin (α-SMA) in human lung sections demonstrated that, in addition to Orai1, hPASMCs also expressed Orai2 and Orai3 in control and patients with PAH (Supplemental Figure 8A). We previously demonstrated by immunoblot experiments that Orai2 protein expression was unchanged in PAH-hPASMCs while Orai3 protein expression was decreased in comparison with control-hPASMCs. As presented in Supplemental Figure 8, D and E, the knockdown of Orai2 or Orai3 had no effect on ER Ca^2+^ release or on SOCE in PAH-hPASMCs. In addition, in PAH-hPASMCs treated with siOrai2 or siOrai3, we found that CM5480 strongly reduced SOCE, demonstrating that SOCE in PAH-hPASMCs is mainly due to Orai1 (Supplemental Figure 8, F and G).

### Orai1 SMC deletion reduced the severity of PAH.

In lung RNA-Seq experiments, we found that CM5480 monotherapy restores some pathways involved in SMC differentiation. We then analyzed the consequences of specific SMC-Orai1 deficiency using unique transgenic rats. We generated conditional Orai1-KO rats targeting specifically SMC using ACTA2 tamoxifen-inducible Cre recombinase (Supplemental Figure 9, A–E). Four weeks after tamoxifen exposure, the quantity of Orai1 protein was reduced by 50% compared with exposure to vehicle (Supplemental Figure 9F). The role of Orai1 in PA tone was assessed using myograph experiments. We found that Orai1 is not involved in contraction evoked by cell membrane depolarization (KCl evoked PA contraction) (Supplemental Figure 9G). Orai1 deficiency induced a significant shift to the right of the dose-response curve for U46619, a thromboxane A2 receptor agonist, indicating that the Orai1 Ca^2+^ channel contributes to PA constriction induced by thromboxane A2 (Supplemental Figure 9H). Three weeks after MCT exposure, we found that inducible specific Orai1 SMC deletion reduced the severity of PAH induced by MCT exposure, as indicated by the reduction of RVSP (Supplemental Figure 9, I–K), confirming that Orai1 dysregulation is a crucial contributor to PAH pathobiology.

### Orai1 pathway is independent of standard PAH-targeted pathways in hPASMCs.

**Figure 6:**
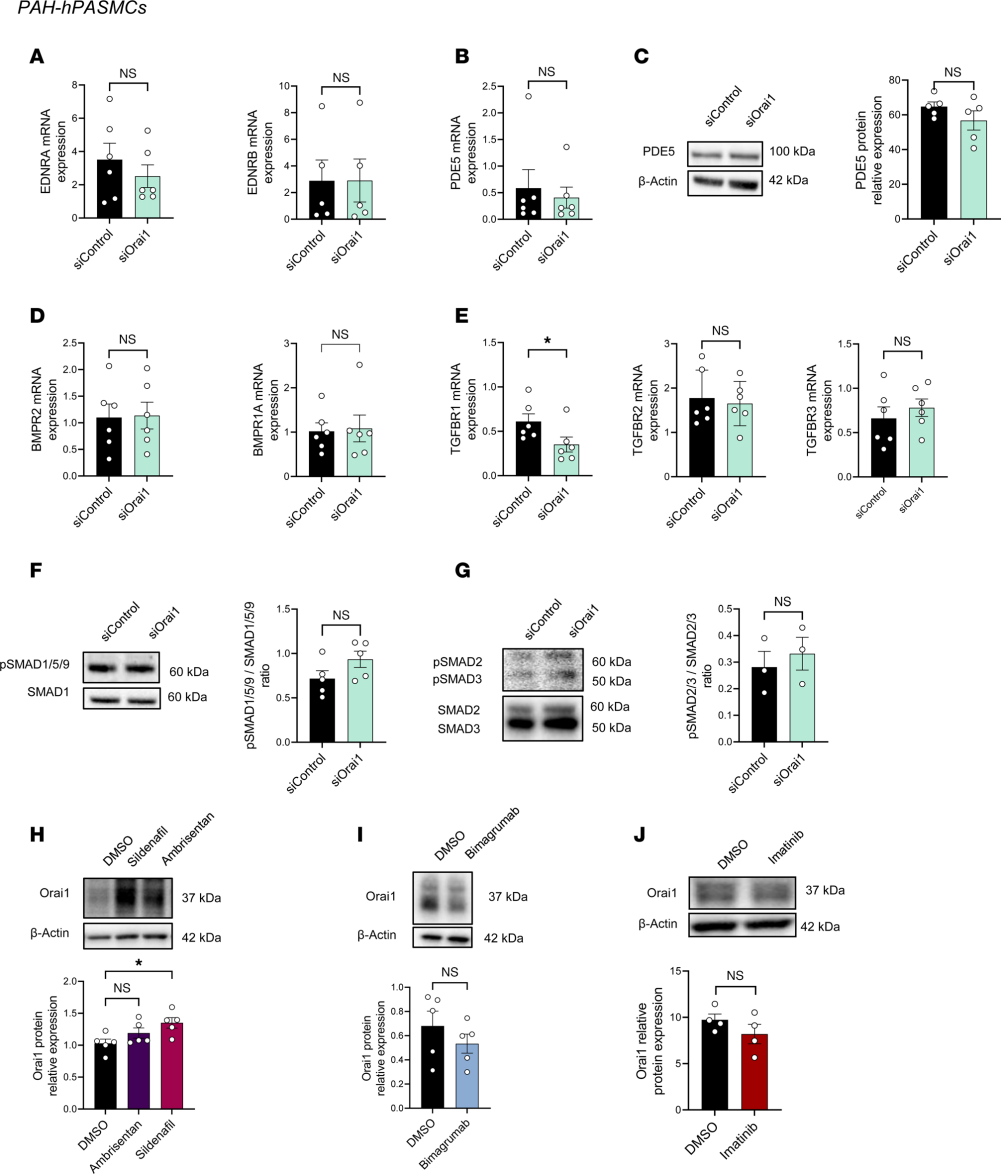
Orai1 pathway is independent of PAH-targeted pathways in PAH-hPASMCs. (**A** and **B**) mRNA expression of EDNRA (*n* = 6 patients), EDNRB (*n* = 5 patients), and PDE5 (*n* = 6 patients). (**C**) Immunoblot images and quantification of PDE5 protein expression in PAH-hPASMCs transfected with siControl or siOrai1 (*n* = 5 patients). (**D**) mRNA expression of BMPR2 (*n* = 6 patients) and BMPR1A (*n* = 6 patients). (**E**) mRNA expression of TGFBR1, TGFBR2 and TGFBR3 (*n* = 6 patients). (**F**) Immunoblot images and quantification of pSMAD1/5/9 / SMAD1 ratio in PAH-hPASMCs transfected with siControl or siOrai1 (*n* = 5 patients). (**G**) Immunoblot images and quantification of pSMAD2/3 / SMAD2/3 ratio in PAH-hPASMCs transfected with siControl or siOrai1 (*n* = 3 patients). (**H**) Immunoblot images and quantification of Orai1 protein expression in PAH-hPASMCs treated with sildenafil (100 μM) and ambrisentan (50 nM) for 48 hours (*n* = 5 patients). (**I**) Immunoblot images and quantification of Orai1 protein expression in PAH-hPASMCs treated with bimagrumab for 48 hours (*n* = 5 patients). (**J**) Immunoblot images and quantification of Orai1 protein expression in PAH-hPASMCs treated with Imatinib (5 μM) (*n* = 4 patients). Mean ± SEM. Two-tailed Student’s *t* test or Mann-Whitney test. *P < 0.05.

Since PAH-hPASMCs are characterized by the overactivation of Orai1 (7), we investigated whether Orai1 interferes with crucial PAH signaling pathways. As presented in Figure 6, Orai1 knockdown did not induce a change in expression of *EDNRA*, *EDNRB* (Figure 6A), or phosphodiesterase 5 (PDE5) mRNA and protein expression (Figure 6, B and C). mRNA expression of *BMPR2*, *BMPR1A*, *TGFBR2*, and *TGFBR3* were unchanged by Orai1 knockdown (Figure 6, D and E), while *TGFBR1* mRNA expression was decreased (Figure 6E). No change in phosphorylation status of SMAD1/5/9 and SMAD2/3 was observed (Figure 6, F and G).

Conversely, we investigated whether approved PAH treatments modulate Orai1 overexpression in PAH-hPASMCs (Figure 6, H–J). The amount of Orai1 protein was increased in PAH-hPASMCs treated with the PDE5 inhibitor (sildenafil). In contrast, an endothelin-1 receptor inhibitor (ambrisentan), a monoclonal antibody inhibitor of activin type II receptors (bimagrumab), and a PDGFR inhibitor (Imatinib) had no consequence on the amount of Orai1 protein expression (Figure 6, H–J).

Aldosterone and mineralocorticoid receptors were demonstrated to contribute to PAH pathogenesis (14) and modulate Orai1 protein expression in cardiomyocytes (15). Nevertheless, we found that treatment with an aldosterone inhibitor (spironolactone 10 μmol/L) or glucocorticoid receptor inhibitors (RU486, 10 μmol/L) did not affect Orai1 protein expression in hPASMCs (Supplemental Figure 10).

These results suggest that the Orai1 pathway did not interfere with the dysregulated pathways already described in PAH-hPASMCs. Alternatively, Orai1 expression is not regulated by these pathways, indicating that Orai1 inhibition is complementary to standard PAH treatments.

### Effect of dual therapy CM5480+ambrisentan or CM5480+sildenafil in comparison with ambrisentan and sildenafil monotherapies in experimental PAH.

**Figure 7:**
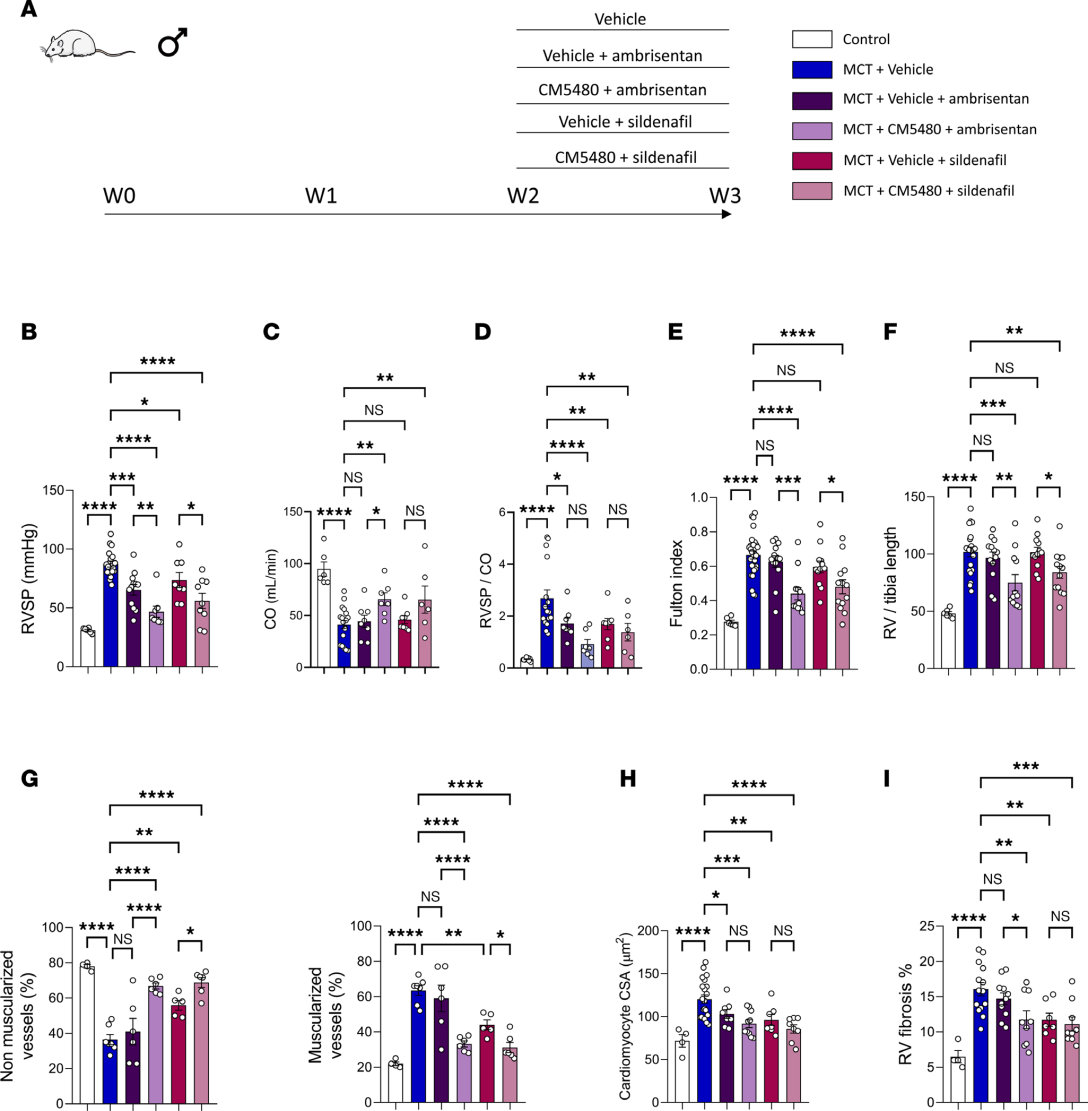
Dual therapy CM5480+sildenafil or CM5480+ambrisentan shows a better efficiency in reducing MCT-induced PAH. (**A**) In vivo experimental design. MCT-rats (male) were treated with vehicle, Vehicle+ambrisentan (10 mg/kg/day), CM5480+ambrisentan (20 mg/kg/day and 10 mg/kg/day, respectively), Vehicle+sildenafil (10 mg/kg/day), or CM5480+sildenafil (20 mg/kg/day and 10 mg/kg/day, respectively), by oral gavage during 7 days between day 14 and day 21. (**B**) RVSP (*n* = 6 for control, *n* = 19 for MCT+Vehicle, *n* = 12 for Vehicle+ambrisentan, *n* = 8 for CM5480+ambrisentan, *n* = 8 for Vehicle+sildenafil, *n* = 9 for CM5480+sildenafil). (**C**) Cardiac output (CO) (*n* = 6 for control, *n* = 19 for MCT+Vehicle, *n* = 8 for Vehicle+ambrisentan, *n* = 7 for CM5480+ambrisentan, *n* = 8 for Vehicle+sildenafil, *n* = 6 for CM5480+sildenafil). (**D**) RVSP/CO (*n* = 10 for control, *n* = 16 for MCT+Vehicle, *n* = 8 for Vehicle+ambrisentan, *n* = 8 for CM5480+ambrisentan, *n* = 8 for Vehicle+sildenafil, *n* = 6 for CM5480+sildenafil). (**E**) Fulton index (*n* = 6 for control, *n* = 28 for MCT+Vehicle, *n* = 13 for Vehicle+ambrisentan, *n* = 12 for CM5480+ambrisentan, *n* = 12 for Vehicle+sildenafil, *n* = 13 for CM5480+sildenafil). (**F**) RV/tibia length (*n* = 6 for control, *n* = 21 for MCT+Vehicle, *n* = 13 for Vehicle+ambrisentan, *n* = 12 for CM5480+ambrisentan, *n* = 12 for Vehicle+sildenafil, *n* = 13 for CM5480+sildenafil). (**G**) Nonmuscularized and muscularized vessels percentage (*n* = 4 for control, *n* = 19 for MCT+Vehicle, *n* = 7 for Vehicle+ambrisentan, *n* = 6 for CM5480+ambrisentan, *n* = 5 for Vehicle+sildenafil, *n* = 6 for CM5480+sildenafil). (**H**) Cardiomyocytes CSA (*n* = 50 cardiomyocytes from 5 rats for control, 21 rats for MCT+Vehicle, 8 rats for Vehicle+ambrisentan, 10 rats for CM5480+ambrisentan, 7 rats for Vehicle+sildenafil, 8 rats for CM5480+sildenafil). (**I**) Quantification of RV fibrosis by Sirius red staining (*n* = 4 for control, *n* = 14 for MCT+Vehicle, *n* = 11 for Vehicle+ambrisentan, *n* = 9 for CM5480+ambrisentan, *n* = 7 for Vehicle+sildenafil, *n* = 9 for CM5480+sildenafil). Mean ± SEM. One-way ANOVA with Fisher’s LSD post hoc test. *P < 0.05, **P < 0.01, ***P < 0.001, ****P < 0.0001.

Our in vitro results suggest that Orai1 inhibition is complementary to endothelin-1 receptor inhibitor (ambrisentan) or PDE5 inhibition (sildenafil) used in standard PAH therapy (Figure 7A). Ambrisentan or sildenafil with monotherapy induced a significant reduction of the elevated RSVP in the MCT rats (Figure 7B) without any consequence on CO, PVR, and RV hypertrophy (Figure 7, C–F). We then determined the putative benefit of dual therapy, CM5480+ambrisentan or CM5480+sildenafil, compared with ambrisentan or sildenafil monotherapy. Compared with their respective monotherapies, the CM5480+ambrisentan or CM5480+sildenafil dual therapy induced a more pronounced reduction in RVSP and PVR and an improvement of CO.

Contrary to ambrisentan or sildenafil monotherapies, CM5480+ambrisentan or CM5480+sildenafil dual therapy strongly reduced RV hypertrophy (Figure 7, E and F). Histological analyses indicate that ambrisentan or sildenafil monotherapies were not significantly beneficial for pulmonary vessel remodeling (Figure 7G) and RV remodeling (Figure 7, H and I), while dual therapies, CM5480+ambrisentan or CM5480+sildenafil, showed improvement of lung vessel remodeling, RV hypertrophy, and RV fibrosis (Figure 7, G–I). In summary, the combined use of CM5480+ambrisentan or CM5480+sildenafil in dual therapy confers more benefits than their respective monotherapies in this preclinical model of PAH.

### Orai1 expression and function are enhanced in pulmonary veno-occlusive disease–lung (PVOD-lung) tissue and SMCs from PA and veins.

**Figure 8:**
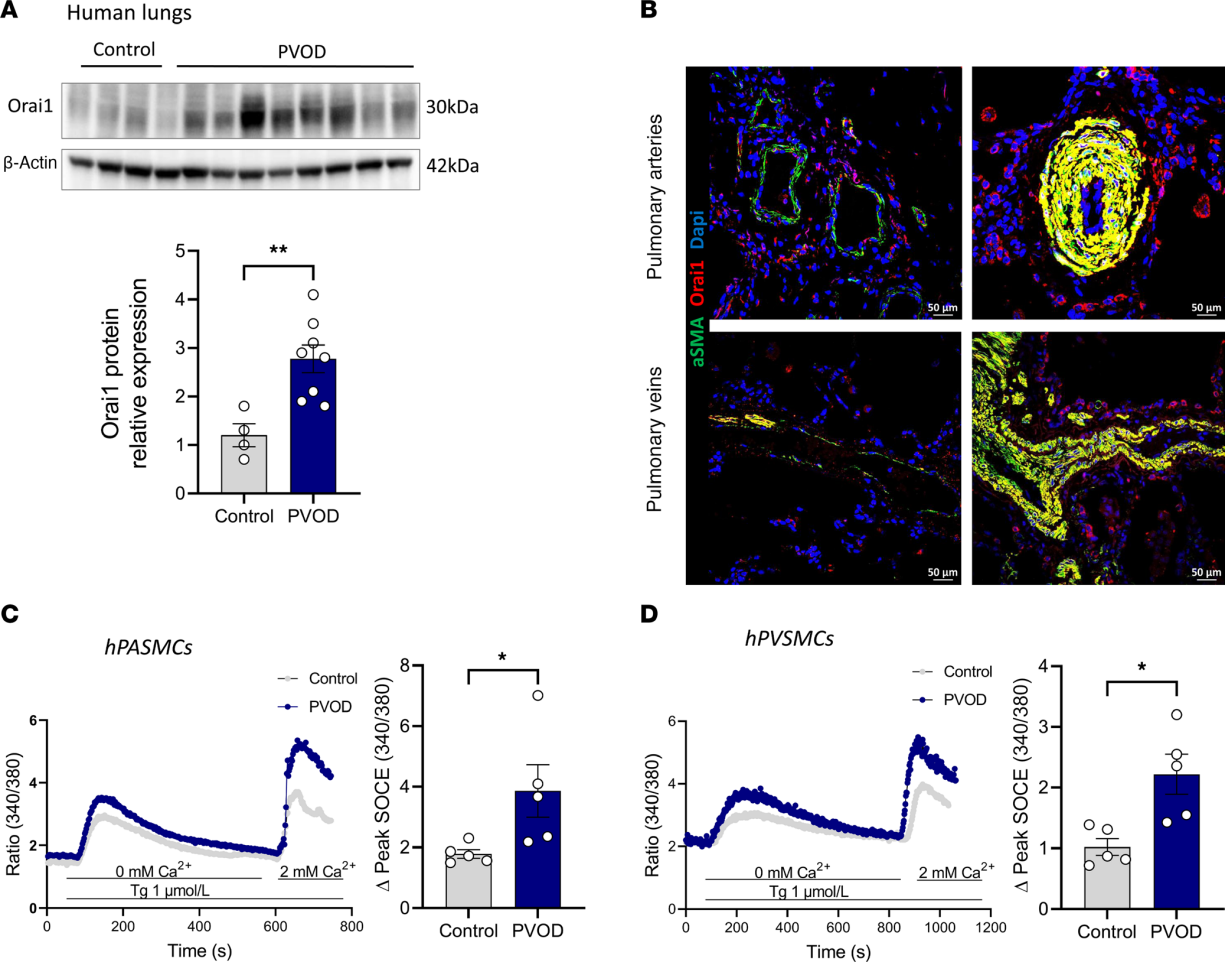
Enhanced Orai1 expression and function in PVOD-hPASMCs and PVOD-hPVSMCs. (**A**) Immunoblot images and quantification of Orai1 protein expression in lungs from control and patients with PVOD (*n* = 4 control and 8 patients with PVOD). (**B**) Localization of Orai1 expression by immunofluorescence staining of paraffin-embedded lung sections from control and patients with PVOD. Orai1 is red, α-SMA is green to localize hPASMCs and hPVSMCs, and nuclei are blue (DAPI). Bar graph = 50 μm. (**C**) SOCE amplitude triggered by Tg (1 μmol/L) on hPASMCs from control and patients with PVOD (*n* = 5 patients for each condition). (**D**) SOCE amplitude triggered by Tg (1 μmol/L) on hPVSMCs from control and patients with PVOD (*n* = 5 patients for each condition). Mean ± SEM. Mann-Whitney *U* test. *P < 0.05, **P < 0.01.

PVOD is a rare form of PAH characterized by a particular remodeling of the pulmonary venous system, lung capillary proliferation, and PA remodeling (16). We assessed the level of expression and function of Orai1 in PVOD. Immunoblot experiments demonstrate higher Orai1 expression in the lungs of patients with PVOD compared with controls (Figure 8A). Orai1 immunofluorescence staining in paraffin-embedded lung sections from control and patients with PVOD demonstrate that Orai1 was localized in hPASMCs and pulmonary vein SMC (hPVSMCs), with an increased Orai1 staining intensity in the remodeled PA and pulmonary veins from patients with PVOD (Figure 8B). The measure of SOCE was performed in control and PVOD-hPASMCs and hPVSMCs. We found an increase in SOCE amplitude in PVOD-hPASMCs and PVOD-hPVSMCs compared with control (Figure 8, C and D).

## Discussion

Despite constant therapeutic innovation for 2 decades, PAH remains a deadly disease. Current therapies are designed to induce PA dilation by targeting endothelin-1 receptor, PDE5, guanylate cyclase, or prostacyclin pathway. Recently, an additional tool in the PAH medical arsenal has become available for patients with PAH. Indeed, sotatercept acts by reequilibrating BMP/TGF-β pathways (17, 18). Because PAH is a complex and multifactorial disease involving several dysregulated signaling pathways, we need to combine different therapeutic strategies to cure PAH. In a recent study, we provided proof of concept that the Orai1 Ca^2+^ channel should be considered as an innovative therapeutic candidate for PAH and heart failure. Commercially available or in-house synthesized Orai1 inhibitors efficiently reduced the development of PAH in rats (7) and LVF in mice (8).

In the present study, we found that the Orai1 blockade strategy is complementary to standard PAH care and could be used in combination therapy with existing PAH therapies. By using CM5480, a selective Orai1 inhibitor from CalciMedica Inc., we found that Orai1 inhibition has the potential to reduce PAH and can be administered in combination with existing therapies. Indeed, the Orai1 blocker Auxora has already been tested in phase II clinical trials for the treatment of acute pancreatitis, severe acute kidney injury (NCT06374797), and severe COVID-19 pneumonia (19–22).

### Role of Orai1 in pulmonary endothelial function.

We previously demonstrated that Orai1 expression and function are unchanged in PAH-hPECs (7). However, in the present study, we show that Orai1 knockdown reduces the cell proliferation and the migration capacity of PAH-hPECs without modifying their capacity to form tubes in vitro. In addition, we found that Orai1 knockdown increases the mRNA expression of *IFNA1*, as well as *PTGFR* and *BMPR2*. It has been shown that *IFNA1* reduces endothelial cell proliferation and migration (23). IFNA receptor 1 inhibition is known to stimulate vascular SMC growth (24). We expected that increased production of IFNA1 by PAH-hPECs in the siOrai1 condition would partly explain the reduced proliferation and migration between PAH-hPASMCs. BMPR2 expression is well described as being downregulated in PAH-hPECs (25), mediating hPECs dysfunction. We found that Orai1 knockdown in PAH-hPECs induces increased BMPR2 expression, suggesting that Orai1 inhibition should partly reduce endothelial dysfunction that occurs in PAH by restoring BMPR2 expression. Recently, Babicheva et al. demonstrated that Orai1 and STIM1-mediated SOCE are involved in the control of endothelium-to-mesenchymal transition-mediated TGF stimulation (26). However, we found that Orai1 knockdown in PAH-hPECs has no effect on the expression of the key transcription factors (SNAI1, SNAI2, TWIST1) involved in the control of epithelial-mesenchymal transition (Supplemental Figure 6, J–L).

### Orai1 and RV remodeling occurring in PAH.

Orai1 expression and function are enhanced in dysfunctional RV cardiomyocytes from MCT-PAH rats (6, 7), as well as in dysfunctional LV cardiomyocytes from LVF mice (8). In LVF mice, Orai1 inhibition restores LV systolic function and Ca^2+^ handling (8). In addition, in isolated RV cardiomyocytes from MCT-PAH rats, in vitro Orai1 inhibition normalizes Ca^2+^ handling and cardiomyocyte contractility (6). In addition, we recently found that the SOCE was enhanced in a large animal model of RV dysfunction in pigs (27). Our RNA-Seq experiments demonstrate that CM5480 treatment restored almost all dysregulated genes induced by the development of RV remodeling and dysfunction, which was not the same in the lung as indicated in lung RNA-Seq experiments. This finding supports the idea that the Orai1 inhibitor CM5480 does not act only on pulmonary vasculature but also on the RV compartment. Further experiments are needed to understand how Orai1 is involved in RV dysfunction occurring in PAH.

### Orai1 inhibitor is suitable for monotherapy or dual therapy in combination with standard PAH care.

In vitro experiments in PAH-hPASMCs suggest that Orai1 overexpression and overactivation are independent of the signaling pathways targeted in PAH, indicating that Orai1 inhibition could be complementary to current therapies. We found that the dual therapies CM5480+ambrisentan or CM5480+sildenafil have beneficial effects in comparison with ambrisentan or sildenafil monotherapy and versus CM5480 alone, by inducing a more pronounced reduction of RVSP and RV hypertrophy, and a more pronounced improvement of CO, PVR, and pulmonary vessels and RV remodeling. Because CM5480, ambrisentan, and sildenafil act via different and complementary mechanisms, these results demonstrate the suitability of CM5480 for use as either monotherapy or in combination with sildenafil or ambrisentan. We also found that the inhibition of activin receptor II B with bimagrumab has no consequence on Orai1 overexpression in PAH-hPASMCs, suggesting that CM5480 monotherapy could be complementary to sotatercept treatment.

### CM5480 and Orai isoforms.

In the present study, our in vitro experiments performed in PAH-hPECs and PAH-hPASMCs indicate that Orai1 is the primary contributor of SOCE, since the knockdown of Orai2 or Orai3 has no effect on SOCE. In addition, the selectivity data from CalciMedica, performed in a heterologous system, indicate that CM5480 is approximately 10 times more potent on Orai1 channels than Orai2 channels. No existing data were collected on the Orai3 channel. It has been recently shown that Orai1 and Orai2 are upregulated in PA and PASMCs from MCT-PAH rats and that Orai1 and Orai2 knockdown, in PASMCs from MCT-PAH rats, reduce SOCE and cell proliferation (28, 29). In systemic artery remodeling induced by endothelial injury, it has been demonstrated that Orai3-mediated Ca^2+^ signaling is upregulated in an animal model of systemic vascular SMCs neointimal remodeling and that in vivo Orai3 knockdown inhibits neointima formation (30, 31). Despite the fact that our in vitro results indicate that Orai1 is the primary actor in pulmonary vascular cells’ dysfunctions in PAH, we cannot exclude that in vivo CM5480 administration in MCT-PAH rats may inhibit Orai2 and/or Orai3, contributing to the benefit of CM5480.

### Effect of Orai1 inhibition with CM5480 in other organs.

Some compounds have undergone clinical trials due to the involvement of Orai1-Ca²^+^ signaling in psoriasis, pancreatitis, asthma, pneumonia, and acute kidney injury. No adverse cardiovascular effects or increased susceptibility to infection have been reported (19, 20, 32, 33). This could be partly explained by the fact that more than 50% inhibition of Orai1 function is required to produce immunosuppressive effects. This is because patients with heterozygous Orai1 and STIM1 mutations exhibit benign phenotypes and with no cardiovascular abnormalities (34). Given its ubiquitous expression and involvement in various diseases, the present study investigated the consequences of chronic administration of CM5480 in control and MCT rats at the histological level. These analyses reveal no significant changes to the architectures of the kidneys, liver, or spleen, confirming the safety of CM5480 in both control and MCT-PAH rats. We also found that in vivo administration of CM5480 does not alter systemic blood pressure, suggesting no change in endothelial or vascular smooth muscle contraction.

### Orai1 inhibitors in clinical trials.

Since the identification of the Orai1 Ca^2+^ channel as the archetype of SOCE (35), Orai1 is now considered a plausible target in several pathological processes, including left heart failure, asthma, pancreatitis, muscular myopathy, inflammation, thrombocytopenia, and PAH. Auxora, the i.v. formulation of the Orai1 selective blocker zegocractin (CM4620) has been tested in several clinical trials (Phase I/II) including studies targeting patients with acute pancreatitis or COVID-19 pneumonia (19, 32). Auxora has shown positive results in treating these diseases, with no reported adverse cardiovascular effects or increased susceptibility to infection. Because we demonstrated in the present study that the Orai1 inhibitor CM5480, or dual therapy with the standard of care for PAH, is effective in reducing PAH at both pulmonary and cardiac levels, it would be very exciting to test Auxora or similar compounds in patients with PAH.

### Orai1 as a key factor of PVOD disease.

PVOD is a rare form of PAH characterized by the narrowing of small pulmonary veins and pulmonary capillary proliferation, as well as PA obstruction (36). We know that PVOD is due to biallelic mutations in *EIF2AK4* (eukaryotic translation initiation factor 2 alpha kinase 4) or induced by exposure to specific chemotherapeutic agents or organic solvents (16). In contrast to PAH, the pathobiological knowledge of PVOD is poor, and patients suffering from PVOD are refractory to standard PAH therapies. Patients with PVOD are associated with poor survival, and PAH therapies do not affect survival (37), indicating that we need to decipher the mechanisms involved in PVOD pathobiology to develop innovative therapeutic strategies. In the present study, we found that Orai1 is localized in hPASMCs and hPVMCs from patients with PVOD, and its expression and function are increased in both vascular cell types compared with control patients. As we know from our and other previous works, Orai1’s increased activity contributes to several pathological phenotypes of PASMC and PVSMCs (38), and these results suggest that targeting Orai1 in PVOD could counteract PA and pulmonary vein remodeling responsible for PVOD.

In conclusion, to our knowledge, CM5480 or a similar compound would represent a first-in-class drug targeting a novel pathway, namely the Orai1 axis. CM5480, used in monotherapy or dual therapy with current existing PAH drug therapies, has the potential to reduce PAH and PVOD. Orai1 inhibitors target a distinct pathway from other PAH drugs and significantly and strongly counteract RV remodeling.

## Methods

### Sex as biological variable.

Our study examined male and female rats, sex-dimorphic effects are reported.

### Data availability.

The authors declare that all supporting data are available within the article and in the Supporting Data Values file. The authors declare that all data supporting the findings of this study are available, to the best of our effort, within this paper and supplemental information files, and will be available from the corresponding authors upon request.

Supplemental Methods are available online with this article. Detailed descriptions of all materials and methods used in this study, including chemicals, animals and surgical procedures, pulmonary vascular cell culture, Orai1 knockdown, antibodies used for Western blots and immunofluorescence experiments, quantitative PCR with specific primers, immunostaining, IHC, histological analysis, intracellular Ca^2+^ measurement, isometric tension measurement, echocardiography, hemodynamic measurements, tissue collection, and statistical analysis are presented in the Supplemental Methods. All raw and processed data have been submitted to the GEO NCBI database with the accession nos. GSE307657 (Lung) GSE307656 (RV).

## Author contributions

ASMW, GR, RA, MG, JS, DM, VC, and FA participated in the research design. ASMW, GR, BM, KEJ, A Boët, RA, A Beauvais, MG, MD, MRG, LT, SM, VDM, VC, and FA conducted the experiments and performed the data analysis. ASMW, GR, JS, DM, VC and FA drafted the manuscript for important intellectual content. ASMW, GR, BM, KEJ, A Boët, RA, MG, A Beauvais, JS, MD, MRG, LT, SM, IA, FB, VDM, SH, CBS, KS, MH, OM, DM, VC, and FA reviewed and revised the final version and approved the manuscript submission.

## Funding support

This work is the result of NIH funding, in whole or in part, and is subject to the NIH Public Access Policy. Through acceptance of this federal funding, the NIH has been given a right to make the work publicly available in PubMed Central.

- French National Institute for Health and Medical Research (INSERM)
- Université Paris-Saclay, the Marie Lannelongue Hospital
- Fédération Française de Cardiologie
- Fondation Maladies Rares, AFM-Téléthon #POCs(2023)-121503
- Fondation Maladies Rares EXM-2019-1006
- Fondation Maladies Rares, Development of experimental models for rare diseases
- BM and ASMW are supported by the Therapeutic Innovation Doctoral School (ED569)
- KEJ is supported by the French association HTAPFrance
- MG is supported by the grant from AFM-Téléthon #POCs(2023)-121503
- RA is funded by the EC Horizon 2020 Marie Skłodowska-Curie grant (No. 847635)

## Supplementary Material

Supplemental data

Unedited blot and gel images

Supporting data values

## Version 1 11/10/2025

Electronic publication
