# Supplementary material for: Combination of Orai1 inhibitor CM5480 with specific therapy mitigates pulmonary hypertension and its cardiac dysfunction
Source: JCI Insight. 2025 Nov 10;10(21):e191780. doi: 10.1172/jci.insight.191780 (PMC12643499; doi:10.1172/jci.insight.191780)
Supplement: Supplemental data [file jciinsight-10-191780-s173.pdf]

Combination of CM5480 (Orai1 inhibitor) with specific therapy mitigates pulmonary hypertension and its cardiac dysfunction

Short title: Orai1 Inhibitor as a new therapy for PAH

Anaïs Saint-Martin Willer[1], Grégoire Ruffenach[1], Bastien Masson[1], Kristelle El Jekmek[1], Angèle Boët[1], Rui Adão[2,3,4,5], Mathieu Gourmelon[1], Antoine Beauvais[1], Jessica Sabourin[6], Mary Dutheil[1], Maria-Rosa Ghigna[1], Laurent Tesson[7], Séverine Ménoret[7,8], Ignacio Anegon[7], Fabrice Bauer[1,9], Vincent De Montpreville[10], Sudarshan Hebbar[11], Carmen Brás-Silva[2], Kenneth Stauderman[11], Marc Humbert[1], Olaf Mercier[1], David Montani[1], Véronique Capuano[1], and Fabrice Antigny[1]

[1]Université Paris-Saclay, INSERM, UMR_S 999, Hypertension Pulmonaire : Physiopathologie and Innovation Thérapeutique (HPPIT), AP-HP, Hôpital Bicêtre, Hôpital Marie Lannelongue (Groupe Hospitalier Paris Saint Joseph), FHU André Cournand, ERN-LUNG, Le Plessis robinson, France (A.SM-W, G.R, B.M, K.EJ, A.B, M.G, A.B, M.D, M-R.G, M.H, O.M, D.M, V.C, F.A)
[2]RISE-Health, Department of Surgery and Physiology, Faculty of Medicine, University of Porto, Alameda Prof. Hernâni Monteiro, Portugal. (R.A, C.B-S)
[3]Department of Pharmacology and Toxicology, School of Medicine, Universidad Complutense de Madrid, Madrid, Spain (R.A)
[4]CIBER Enfermedades Respiratorias (Ciberes), Madrid, Spain (R.A)
[5]Instituto de Investigación Sanitaria Gregorio Marañón (IiSGM), Madrid, Spain (R.A.)
[6]Inserm, UMR-S 1180, Signalisation et Physiopathologie Cardiovasculaire, Université Paris-Saclay, Orsay, France. (J.S)
[7]INSERM, Nantes Université, CHU Nantes, Center for Research in Transplantation and Translational Immunology, UMR 1064, Nantes, France. (L.T., S.M., I.A.).
[8]Nantes Université, CHU Nantes, INSERM, CNRS, SFR Santé, Inserm UMS 016 CNRS UMS 3556, Nantes, France (S.M).
[9]Cardiology Department, Bicêtre University Hospital, Le Kremlin-Bicêtre, France (F.B)
[10]Department of Pathology, Groupe Hospitalier-Marie Lannelongue, Le Plessis-Robinson, France (V.D-M)
[11]CalciMedica Inc., La Jolla, California, USA. (S.H, K.S)

Corresponding author: Fabrice Antigny, INSERM UMR_S 999, Hôpital Marie Lannelongue, 133, Avenue de la Résistance, F-92350 Le Plessis Robinson, France. Tel.: (33) 1 40 94 22 99; E-mail: fabrice.antigny@inserm.fr

MATERIAL AND METHODS

Sex as biological variable: Our study examined male and female rats, sex-dimorphic effects are reported.

Chemical: Monocrotaline (MCT), thapsigargin (Tg), spironolactone (S3378), RU486 (M8046) and cytosine arabinoside were obtained from Sigma. Sildenafil (UK-92480, HY-15025), ambrisentan (HY-13209), imatinib (HY-15463) and bimagrumab (HY-P99355) were obtained from MedChemExpress. Fura-2-am and TRIzol reagents were obtained from ThermoFisher Scientific. U46619 was obtained from R&D Systems.

Human PASMCs, PVSMCs and PECs isolation and culture maintenance: hPASMCs hPVSMCs and hPECs were isolated from pulmonary arteries or pulmonary veins obtained during lung transplantation for PAH patients and lobectomy or pneumonectomy of localized lung cancer from control subjects. Pulmonary arteries were excised at a distance from tumor areas.

hPECs were isolated by digesting the lung with dispase (1,7 U/mg) for 2h at 37°C, under agitation, and then filtered at 70 µm. After a few days, hPECs were selected by using CD31 magnetic beads (Dynabeads CD31 Endothelial cell, Invitrogen). hPECs were maintained in MCDB (Gibco), 10% FCS, 2 mM L-Glutamine, 25 mM HEPES, 100 U/mL penicillin-streptomycin, 6 µg/mL ECGS, 1 ng/mL VEGF, 1 U/mL heparin at 37°C, 5% CO_2.

hPASMCs were isolated by removing adventitia from the pulmonary artery. The pulmonary artery or the pulmonary vein were opened, and the intern face is placed on a 6-well plate, and DMEM was added. When cells migrated from the artery or vein, they were amplified. Cells were cultured in DMEM (Gibco) supplemented with 12,5% FCS, 2 mmol/L L-Glutamine, 1X Insulin-Transferin-Selenium, 100 U/mL penicillin-streptomycin, 20 mmol/L HEPES, 10 ng/mL EGF. Cells were used between passages 2 and 5. hPVSMCs were isolated from pulmonary vein and cultured as hPASMCs.

Preparation of CM5480, sildenafil, and ambrisentan for in vivo use: CM5480 spray dried dispersion used for oral administration was produced and provided by CalciMedica. The powder was suspended in a vehicle solution 0.5% methylcellulose (Sigma-Aldrich) and 1% Tween 80 (Sigma-Aldrich) (w/w) in water. The dose volume was 10 mL/kg and dose level 20 mg/kg. Sildenafil was dissolved in 10% DMSO and then in 90% of the vehicle solution (0.5% methylcellulose +1% Tween 80). Ambrisentan was dissolved in the vehicle solution (0.5% methylcellulose +1% Tween 80).

In vivo experiments:

Wistar rats (4 weeks old from Janvier Labs) were used in three experimental protocols:

1. PAH was induced by a single MCT injection (60 mg/kg, s.c.) in male rats. MCT was dissolved in 1 N HCl and neutralized with 1 N NaOH. Control animals received the same volume of saline solution. MCT-exposed rats were treated with CM5480 (20 mg/kg/day in vehicle, daily by oral gavage) from week 1 (W1) to week 3 (W3). CM5480 was prepared in its vehicle as described above. Control animals received the same volume of vehicle.
2. PAH was induced by a single MCT injection (60 mg/kg, s.c.) in male and female rats. MCT was dissolved in 1 N HCl and neutralized with 1 N NaOH. Control animals received the same volume of saline solution. MCT-exposed rats and control rats were treated with CM5480 (20 mg/kg/day in vehicle, daily by oral gavage) from week 2 (W2) to week 3 (W3). CM5480 was prepared in its vehicle. Control animals received the same volume of vehicle.
3. PAH was induced by a single MCT injection (60 mg/kg, s.c.) in male rats. MCT was dissolved in 1 N HCl and neutralized with 1 N NaOH. Control animals received the same volume of saline solution. MCT-exposed rats were treated with sildenafil (10 mg/kg/day in vehicle, daily by oral gavage) or ambrisentan (10 mg/kg/day in vehicle

by oral gavage) or sildenafil+CM5480 (20mg/kg/day and 10 mg/kg/day respectively by oral gavage) or ambrisentan+CM5480 (20 mg/kg/day and 10mg/kg/day respectively by oral gavage) from week 2 (W2) to week 3 (W3). CM5480 was prepared in its vehicle. Control animals received the same volume of vehicle.

Generation of Orai1 inducible knockout rats.

All animal experiments were approved by the Animal Experimentation Ethics Committee of the Pays de la Loire region, France, in accordance with the guidelines from the French National Research Council for the Care and Use of Laboratory Animals (permit number: Apafis 26567) and supervised in compliance with the European Community guidelines for laboratory animal care and use and every effort was made to minimize the number of animals used and their suffering.

The strategy to generate an inducible knockout of *Orai1* in cells expressing Acta2 was to generate two rat lines that would be crossed. One with 2 loxP sites introduced at the 5' and 3' side of the last exon of *Orai1*. The second line would harbor a Cre-ERT2/RFP cassette at the 3' side of the last exon of the *Acta2* locus which results in Cre-ERT2/RFP expression restricted in cells expressing Acta2. When these two lines will be crossed, animals would carry both Cre-ERT2/RFP and floxed *Orai1*. To remove *Orai1* expression, Tamoxifen (20mg/kg) will be given to rats to eliminate exon 7 and the termination codon *Orai1* which would lead to a inducible knockout in *Acta2* expressing cells (smooth muscle cells).

The two rat lines were generated by using the CRISPR/Cas9 technology. To flox the *Orai1* exon, sgRNA were designed, produced and tested by GenoCellEdit platform (GCE, Nantes, France). The sg RNA G58-rOrai1-sg165R targeting the following sequence: CCACCTTGACTATTCATGAT and the sg RNA G60-rOrai1-sg157R targeting the following sequence: AGTTGTAGGACTAATCCCAG were in vitro transcribed with the HiScribe T7 high yield RNA Synthesis kit (New England Biolabs) and were purified using EZNA

microelute (Omega Bio-Tek, VWR). The donor DNA *Orai1*-loxP-LastExon-loxP was synthetized by GeneCust (Boynes) and prepared for microinjection by TRIP platform. To introduce the ACTA2-CreERT2RFP cassette, the sgRNA to target ROSA locus was designed, produced and tested by the GCE platform. The sgRNA ROSA-G78 target the following sequence: GTTTCATACACCACAAATCG was in vitro transcribed with the HiScribe T7 high yield RNA Synthesis kit and were purified using EZNA microelute. The donor DNA HAROSA-ACTA2-CreERT2RFP was produced and prepared for microinjection by TRIP platform with T2ARFP fragment synthetized by GeneCust and the ACTA2 promoter was kindly provided by Pr OWENS GK (Charlottesville, VA, USA).

These sgRNA (0.2µg/µl) and Cas9 protein (3µmol/L) were incubated at room temperature for 10 min to allow the formation of RNP complexes and DNA donor (2ng/ml) was then added and then were kept at 4°C until microinjection. Embryo obtention and microinjection were performed as previously described (1). Briefly, zygotes were collected from pre-pubescent (4–5-week-old) donor female rats (Wistar strain, Janvier Labs), superovulated by injection of pregnant mare serum gonadotropin (30 IU, i.p.; Centravet) and, 48h later of human chorionic gonadotropin (20 IU; Centravet) and then mated with fertile males. One-cell-stage fertilized embryos were collected in the Transgenic Rat ImmunoPhenomic platform and sequentially microinjected into the male pronucleus and into the cytoplasm. Microinjected zygotes were maintained under 5% CO_2 at 37°C for 2h. Surviving embryos were implanted on the same day in the oviduct of pseudo-pregnant females (0.5 dpc) and allowed to develop to full term.

Genotyping.

For PCR and sequence analysis, ear biopsies were included in the Proteinase K lysis buffer for PCR testing. Primers used for rOrai1_LoxP knock-in rats genotyping: PCR-based genotyping for 5’ exon 7/NcoI-loxP forward primer BLER-3-seq: 5’-AGTTTGCTCCCTAGAACCTACA-3’ and BLER: 5’-TGTGTGTCCCAGCCCTTATAGA-3’. PCR-based genotyping for 3’ exon

7/loxP-BamHI forward primer: V-sg43-2S-Fw: 5'-TCTTCCTCATGCCTGTGTTTGT-3' and V-sg43-2S-Rev: 5'-CAAACCACATGCCTTCCCAG-3'. PCR was performed using a proofreading DNA polymerase (Herculase II fusion enzyme kit, Agilent technologies) and a standard thermocycler (Veriti, Applied Biosystems, Waltham). The PCR conditions were as follow: initial denaturation (95 °C for 2 min), followed by 35 cycles of denaturation (95 °C for 10 s), annealing (60 °C for 10 s) and extension (72 °C for 30s) and a final extension at 72 °C for 3 min. PCR amplicons were direct digested by NcoI enzyme or BamHI enzyme (New England Biolabs). Primers used for line rActa2-CreERT2/ROSA knock-in rats genotyping: PCR-based genotyping CreERT2-2184 For1 primer: 5'-ACCAGGTTCGTTCACTCATGGA-3' and CreERT2-3235 Rev1 primer: 5'-GGAACCGAGATGATGTAGCCAGC-3'. PCR-based genotyping 3prACTA2promFor primer: 5'-CGCTCCTGCTCTTTGTGCTGA-3' and CreERT2-2277Rev primer 5'-TGATCCTGGCAATTTCGGCTA-3'. The PCR conditions were as follows: initial denaturation (95 °C for 5 min and 60°c for 2min), followed by 35 cycles of annealing (72 °C for 40 s), denaturation (95 °C for 10 s) and extension (58 °C for 60s) and a final extension at 72 °C for 3 min and sequencing. Capillary electrophoresis results were analyzed using LabChip GX software (Perkin Elmer), as previously described (2).

Echocardiographic measurements: Evaluation by transthoracic echocardiography (TTE) was performed with a digital ultrasound system (Vivid E9, GE Healthcare) using a high frequency phased array transducer (12 S-D 4-12 MHz, GE Healthcare). The echocardiographic evaluation procedure was performed under general anesthesia and spontaneous breathing with an Isoflurane Rodent Anaesthesia System (Minerve) (maintenance isoflurane 2% at room air). Rats were shaved, and body temperature was controlled during experiments. All analyses were blinded: the experimental condition of each rat was unknown by the operator during TTE examination and data interpretation (3).

Hemodynamic measurements and tissue collection: Rats were placed under general anesthesia and spontaneous breathing with an isoflurane Rodent Anesthesia System (Minerve). Hemodynamic measurements, such as heart rate (HR), right ventricular (RV) systolic pressure (RVSP; mmHg), and cardiac output (CO; mL/min), were blindly measured in unventilated anesthetized rats using a closed-chest technique. Under profound anesthesia, animals were euthanized by exsanguination. After catheterization, tissues were collected, and Fulton index (a measure of RV hypertrophy) was calculated as the ratio of RV weight to left ventricular (LV) plus septum (S) weight: RV ÷ (LV + S) (3).

Isometric tension analysis: Contractile response of rat pulmonary arteries (PA) was assessed by using a small-vessel wire myograph (EMKA Technology) coupled with IOX software (EMKA Technologies). As determined in preliminary experiments, rat PAs were set at an optimal length by equilibration against a passive load of 0.3 g. Vessels were bathed in Krebs solution containing (in mmol/L) 119 NaCl, 4.7 KCl, 2.5 $CaCl_2$, 1.17 $MgSO_4$, 1.18 KH_2PO_4, 25 NaHCO3, and 11 glucose at 37°C, and continuously aerated with a mixture of CO_2/O_2 (5%/95%). After adding Krebs solution, vessels were contracted with 100 mmol/L K^+-containing solutions (K100). Once a plateau was reached, the vessels were washed with Krebs solution for 30 min, and then dose response to KCl (10–90 mmol/L) or dose-response to U46619 (0.1 nmol/L–3 µmol/L) was applied. All modified Krebs solutions prepared with various KCl concentrations (K10 to K100) had an equimolar substitution of NaCl to maintain a constant osmolarity and $[Cl^-]$ compared with standard Krebs solution. The contractile response was expressed relative to the response to the K100 challenge.

Western blot analysis: Total protein from tissue or cell samples from rats or humans were prepared in lysis buffer containing 1% Igepal, 20 mmol/L Tris HCl, 137 mmol/L NaCl, 10% Glycerol, 2 mmol/L EDTA, 1 mmol/L Na_3VO_4, leupeptin 10 µg/µl, pepstatin 10 µg/µl, aprotinine 10 µg/µl and protease inhibitor cocktail (aprotinin, leupeptin, and PefaBloc [Roche]).

Protein lysates (40 μg for tissue or 20 μg for cells) were separated by SDS-PAGE and transferred to a nitrocellulose or PVDF membrane. After blocking, membranes were incubated in T-TBS and 5% BSA overnight at 4°C with primary antibodies.

Blots were incubated with HRP conjugated goat anti-mouse diluted 1:10000 (Cell Signaling) or with HRP-conjugated goat anti-rabbit diluted 1:5000 (Cell Signaling), respectively. Antibodies were revealed using Amersham ECL reagents (Cytiva) under the imaging system ChemiDoc XRS+, BioRad, coupled with ImageLab Software 6.1. β-actin was used as a loading control. ImageJ Software was used to quantify the level of protein expression. The list of antibodies used is presented in Supplemental Table 1.

Immunofluorescence staining: Lungs were fixed in 4% paraformaldehyde, paraffin-embedded, and serially sectioned (5 μm). The tissue sections were deparaffinized in xylene baths before rehydration in alcohol. Epitope unmasking was performed in a pH 9 buffer using a 2100 Antigen Retriever (Aptum). The slides were saturated with 0.5 M NH_4Cl for 15 minutes, then blocked and permeabilized for one hour at room temperature with a solution containing 1% BSA (Bovine Serum Albumin), 0.5% Triton X100, 10% human serum, and 10% donkey serum. The primary antibody against Orai1, Orai2 or Orai3 and anti-vWF (1/200) antibody or anti-alpha-SMA-FITC antibody were incubated overnight at 4°C in a solution of PBS, 0.2% BSA, 0.2% Triton X100, and 3% donkey serum. The slides were then incubated for 1h in the dark at room temperature with a secondary antibody (1/400) donkey anti-rabbit Alexa Fluor 594 (to detect Orai1, Orai2 or Orai3) and donkey anti-mouse Alexa 647 (to detect vWF) with 3% human serum and DAPI. Antibodies used for immunofluorescence experiments are summarized in Supplemental Table 2.

Transcriptomic analysis:

RNA extraction and purification: Total RNA was extracted from lung tissues using TRIzol (Invitrogen) with DNase digestion. RNA concentration and purity were evaluated on a

NanoDrop (Thermo Scientific) spectrophotometer measuring absorbance at 230, 260, and 280 nm. RNA integrity was controlled on a Bioanalyzer 2100 using the RNA 6000 Nano kit (Agilent Technologies, USA). RNA concentration was measured on a Xenius (Safas) fluorimeter using the Quantifluor® RNA System kit (Promega).

Transcriptome analysis by RNA sequencing: Directional RNA-Seq Libraries were constructed from 1 µg of total RNA using the TruSeq Stranded mRNA Library Prep kit (Illumina), following the manufacturer's instructions. Final libraries were qualified on a Bioanalyzer 2100 using the High Sensitivity DNA Kit (Agilent Technologies), and library concentration was measured on a Xenius fluorimeter (Safas) using the PicoGreen™ kit (Invitrogen). Libraries were pooled in equimolar proportions and sequenced in one 100-bp single read P2 run on a NextSeq 2000 instrument (Illumina) by the I2BC High-throughput sequencing facility (Gif-sur-Yvette, France).

RNA-seq data analysis: All analysis was performed using R (https://www.r-project.org/) and RStudio (http://www.rstudio.com/) as previously described in (4).

All RNA-seq data obtained in lung and RV tissues are in Supplemental Table 4 and 5, respectively. All raw and processed data have been submitted to the GEO NCBI database with the accession numbers GSE307657 (Lung) GSE307656 (RV).

Reverse Transcriptase Quantitative Polymerase Chain Reaction (RT-qPCR): Total RNA from hPASMCs or hPECs was isolated by using TRIzol reagent. RNA concentration and purity were evaluated on a NanoDrop (Thermo Scientific) spectrophotometer measuring absorbance at 230, 260, and 280 nm. RT was performed with a StaRT kit from AnyGenes (Paris, France) according to the manufacturer's protocol, and qPCR was performed with perfect Master Mix SYBR Green (with ROX) for SignArrays 96 system from AnyGenes according to the manufacturer's protocol on a Plus Real-Time PCR System (Life Technologies) and analyzed with StepOne Software. For PAH-hPECs, Endothelial Dysfunction plaques (PZ75A1H1-F)

were used. A special design was made to study the dysfunction of hPASMCs. All the primers used are in Supplemental Table 6 for hPECs and Supplemental Table 7 for hPASMCs.

For siRNA validation, RT was performed with QuantiTect Reverse Transcription (Qiagen) according to manufacturer's protocol on a Thermal Cycler T100 (Bio-Rad). qPCR were performed with TaqMan gene expression assays (Life Technologies) on a StepOne Plus Real-Time PCR system (Life Technologies). Primers used for siRNA validation are summarized in Supplemental Table 8.

siRNA transfection: hPASMCs and hPECs were transfected in suspension by incubating $4x10^5$ cells in a solution containing 1 mL of Opti-MEM (Gibco, ref 31985-062), 6 µL of Lipofectamine RNAiMax (Invitrogen, ref 13778-150) and 50 nmol/L of a specific Silencer™ Select siRNA against Orai1, Orai2 or Orai3 (siOrai1, #s99509; siOrai2, #s199503; siOrai3, #s41088, Thermo Fisher Scientific) or Silencer™ Select negative control (siControl). All experiments were performed 72 hours after transfection. The Orai1 knockdown efficacy has already been validated in our hands in hPASMCs and hPECs (3).

Proliferation assay: To assess the proliferation capacity of PAH-hPECs, we quantified BrdU incorporation in cells undergoing DNA replication using a DELFIA cell proliferation kit (AD0200, PerkinElmer). Experiments were performed according to the manufacturer's protocol. siRNA was used in these experiments. Cells were starved for 48h by using a medium containing 2% SVF 24h hours after transfection.

To evaluate the paracrine effect of PAH-hPECs transfected with siOrai1 or siControl, we collected hPECs medium 72h after transfection and put it on control-hPASMCs for 24h with BrdU.

The fluorescence signal in the 96-well plate was read with a FlexStation3 (Molecular Devices).

Cell Migration assay: A wound healing assay was performed to assess cell migration. PAH-hPECs were plated in a 2-wells culture insert (Cat. No. 80209; Ibidi) 24h after transfection at a density of 2 x 10^4 cells per well in a fresh medium with cytosine arabinoside (10 µmol/L). After allowing the cell to attach, culture inserts were removed, and cells were washed with 1X PBS to remove non-adherent cells. Fresh medium was added, and a picture of time 0 and time 8 hours was taken using an EVOS microscope (Invitrogen) at 4X. Quantification of wound closure was performed on ImageJ using a macro (MRI Wound Healing Tool). The percentage of wound healing closure 8 hours after initiation of wound healing was calculated as follows: ([(area T0 − area T8) ÷ area T8] × 100).

In vitro tube formation assay: 96-well plates were precoated with extracellular matrix gel provided in the Endothelial Tube Formation Assay kit from Cell Biolabs. 72 hours after control-hPECs transfection, 5 × 10^3 cells/well were seeded onto the gel. Cells were incubated for 4 hours at 37 °C in the standard culture medium, and tubular network visualization was performed after staining cells with Calcein/AM dye (10 µmol/L) using EVOS (Invitrogen). Tube formation was quantified as the number of junctions and segments, and the total master segment length was analyzed as previously described (5).

Intracellular Ca^{2+} measurements: hPECs, hPASMCs and hPVSMCs were plated on 18-mm glass coverslips and loaded with 2 µmol/L Fura-2-AM dissolved in DMSO plus 20% pluronic acid (Life Technologies) and then incubated at 37°C for 30 min in darkness in Krebs solution (in mmol/L:135 NaCl, 5.9 KCl, 2 $CaCl_2$, 1 $MgCl_2$, 10 HEPES, 10 D-glucose) at pH 7.4. Loaded cells were washed twice with the physiological solution before imaging. Ca^{2+} images were obtained using a microscope (Olympus IX71) equipped with a Sutter Fluo Lambda 421 LED system (Sutter Instrument Company), which rapidly changed the excitation wavelengths between 340 nm and 380 nm. Emission was measured at 510 nm. The Ca^{2+}-free solution contained 1 mmol/L EGTA instead of 2 mmol/L $CaCl_2$ + nifedipine (L-Type Ca^{2+} channel

inhibitor) + KB-R7943 (Na^+/Ca^{2+} exchanger inhibitor). SOCE was triggered by Thapsigargin (Tg, 1 µmol/L) stimulation. Calcium signaling was also recorded after histamine (10 µmol/L) stimulation. The area under the curve was analyzed with GraphPad Prism 8. Due to the high transfection efficiency, cytosolic Ca^{2+} measurements were performed on the whole cell population. Image acquisition in selected cells and analysis was performed with MetaFluor® 7.8 imaging software (Molecular Devices) (3).

Histological assessment on hearts: Hearts were fixed in 4% paraformaldehyde, paraffin-embedded, and serially sectioned (5 µm). Heart sections were stained with Sirius red to assess heart fibrosis. Pictures were recorded at 10X on an EVOS microscope (Invitrogen). RV fibrosis was quantified with ImageJ software. To quantify cardiomyocyte hypertrophy, heart sections were stained with FITC-conjugated wheat germ agglutinin (WGA) (50 µg/mL). Pictures were taken on an EVOS microscope (Invitrogen) at 40X. RV cardiomyocytes' diameters were determined using Image J software by measuring the short axis of 10 cells/field (5 fields/ventricle/animal).

Lung, kidney, spleen and liver histological assessment: Kidney, spleen and liver were fixed in 4% paraformaldehyde, paraffin-embedded, and serially sectioned (5 µm). Kidney, spleen and liver sections were stained with Hematoxylin-Eosin-Saffron (HES).

Lung sections were immunostained with anti-α-SMA-FITC and anti-vWF (Rabbit Dako, A0082) and anti-rabbit Alexa 594 to access the number of non-muscularized and muscularized arteries by counting 100 arteries for each rat. Muscularized vessels are α-SMA-FITC and von Willebrand factor positives, while non-muscularized vessels are only von Willebrand factor positives.

In vitro studies in isolated skinned cardiomyocytes: RV samples, stored at -80°C, were defrosted in a relax solution. A biopsy was taken, subjected to mechanical disruption, and then permeabilized with 0.1% Triton X-100. Under microscopic observation (model 1X51,

Olympus) and using imaging software (VSL 900B, Aurora Scientific), a single cardiomyocyte was attached to a force transducer (model 403A, Aurora Scientific) and a length controller (model 315C-I, Aurora Scientific). Cell length was digitally adjusted using custom-designed software (series 600A digital controller, Aurora Scientific). Steady-state passive force at increasing sarcomere lengths (from 1.8 to 2.3 µm) was measured, and maximal force development was recorded at 2.2 µm (6).

Statistics : Analyses were performed using GraphPad Prism software (GraphPad, version 9.0 for Windows) and R statistical computing software. After checking the distribution of our samples with the Shapiro–Wilk Test, differences between the two or more groups were assessed using an unpaired t test or one-way ANOVA. When conditions of parametric tests were not met, we used either a Mann–Whitney test or Kruskal–Wallis test. All values are reported as means ± SEM. Representative images/figures were chosen to represent the mean of each quantification. For all experiments, a p-value of <0.05 was considered statistically significant.

Study approval: Patients studied were part of a program approved by our institutional Ethics Committee and had given written informed consent (ID RCB: 2018-A01252-53). Patients were part of the French Network on Pulmonary Hypertension, a program approved by our institutional ethics committee, and provided written informed consent (Protocol N8CO-08- 003, ID RCB: 2008-A00485-50). All human tissues were obtained with written informed consent from transplant recipients or families of organ donors in accordance with the Declaration of Helsinki.

The animal facility is licensed by the French Ministry of Agriculture (agreement N° C92-019-01). This study was approved by the Committee on the Ethics of Animal Experiments (CEEA26 CAP Sud). Animal experiments were performed in accordance with the guidelines of Directive 2010/63/EU on September 22nd, 2010, of the European Parliament on the protection of animals

used for scientific purposes and complied with the French institution's guidelines for animal care and handling (project # 42114 and #35323).

References

1. Ménoret S, et al. Homology-directed repair in rodent zygotes using Cas9 and TALEN engineered proteins. *Sci Rep*. 2015;5:14410.

2. Chenouard V, et al. A Rapid and Cost-Effective Method for Genotyping Genome-Edited Animals: A Heteroduplex Mobility Assay Using Microfluidic Capillary Electrophoresis. *J Genet Genomics Yi Chuan Xue Bao*. 2016;43(5):341–348.

3. Masson B, et al. Orai1 Inhibitors as Potential Treatments for Pulmonary Arterial Hypertension. *Circ Res*. 2022;101161CIRCRESAHA122321041.

4. Ruffenach G, et al. Transcriptome analyses reveal common immune system dysregulation in PAH patients and Kcnk3-deficient rats. *Pulm Circ*. 2024;14(4):e12434.

5. Ribeuz HL, et al. Role of KCNK3 Dysfunction in Dasatinib-associated Pulmonary Arterial Hypertension and Endothelial Cell Dysfunction. *Am J Respir Cell Mol Biol*. 2024;71(1):95–109.

6. Hautefort A, et al. Bmpr2 Mutant Rats Develop Pulmonary and Cardiac Characteristics of Pulmonary Arterial Hypertension. *Circulation*. 2019;139(7):932–948.

Supplemental Table 1: Antibodies used for immunoblotting experiments.

Antibody	Species	Dilution	Supplier	Reference
Orai1	Rabbit	1/2000	Sigma-Aldrich	O8264
Orai2	Rabbit	1/1000	Alomone	ACC-061
Orai3	Rabbit	1/1000	Alomone	ACC-065
PDE5	Rabbit	1/1000	Abcam	Ab64179
pSMAD1/5/9	Rabbit	1/1000	Cell Signaling	13820
SMAD1	Rabbit	1/1000	Cell Signaling	6944
pSMAD2/3	Rabbit	1/1000	Cell Signaling	8828
SMAD2/3	Rabbit	1/1000	Cell Signaling	8685
β-actin-HRP	Mouse	1/3000	Santa Cruz Biotechnologies	Sc-47778

Supplemental Table 2: Antibodies used for immunostaining experiments.

Antibody	Species	Dilution	Supplier	Reference
Orai1	Rabbit	1/100	Sigma-Aldrich	O8264
Orai2	Rabbit	1/100	Alomone	ACC-061
Orai3	Rabbit	1/100	Alomone	ACC-065
α-SMA-FITC	Mouse	1/200	Sigma-Aldrich	F3777
vWF	Mouse	1/200	Sigma-Aldrich	AMAB9091

Supplemental Table 3: Effect of preventive inhibition of Orai1 with selective inhibitor CM5480 in male MCT-rats.

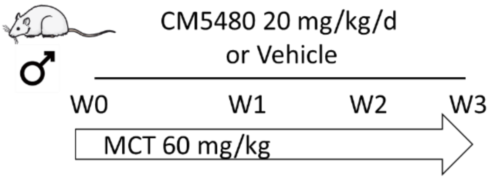

	MCT-vehicle (mean ± SEM)	MCT-CM5480 (mean ± SEM)	P-value	Significance
PAAT	24.5±1.24	28.38±1	0.0291	*
RVET (ms)	70.00±2.85	79.00±1.77	0.0138	*
VTI PA	4.23±0.45	5.34±0.44	0.0469	*
RV thickness (mm)	1.56±0.09	1.34±0.08	0.0884	ns
RVesD	2.29±0.22	2.44±0.21	0.5737	ns
RVedD	3.80±0.21	3.62±0.16	0.5737	ns
RV FS%	33.57±5.12	33.05±4.25	>0.9999	ns
RVSP (mmHg)	92.63±4.95	57.81±2.45	0.0003	***
Min dP/dt (mmHg/s)	-3768±163.4	-3044±97.11	0.0093	**
Max dP/dt (mmHg/s)	3869±144.5	2972±118.4	0.0012	**
Conctractility index (1/s)	105.8±8.59	127.5±7.61	0.1206	ns
Cardiac output (mL/min)	51.22±7.72	70.80±5.56	0.0973	ns
PVR	2.25±0.51	0.95±0.13	0.0037	**
Fulton index	0.50±0.05	0.33±0.02	0.0005	***
RV/tibia lenght	8.83±0.53	6.22±0.45	0.0044	**
RA/LA	3.89±0.46	2.28±0.21	0.0092	**
RA/total weight	0.37±0.04	0.22±0.02	0.051	**
Carotid artery mean pressure (mmHg)	93.03±3.75	90.79±6.22	0.9458	ns
VTI Aorta	4.63±0.45	6.25±0.25	0.0062	**
LVedD	5.64±0.24	6.37±0.20	0.0303	*
LVesD	3.57±0.30	3.75±0.17	0.3667	ns
LV FS%	36.94±4.07	40.82±2.81	0.5737	ns
E/A	1.45±0.20	1.92±0.06	0.0650	ns
LV thickness (mm)	1.23±0.06	1.18±0.06	0.7781	ns
LV/tibia lenght	18.08±0.68	17.29±1.79	0.5925	ns

T-test or Mann-Whitney test. ns = non significant; * P<0.05, **P<0.01, ***P<0.001.

Supplemental Table 6: Primers used in RT-qPCR for hPECs.

Gene name	RefSeq	Symbol	Forw Primer location	Rev Primer location	Amplicon size (bp)
intercellular adhesion molecule 1	NM_000201.2	**ICAM1**	exon 5	exon 6	77
platelet and endothelial cell adhesion molecule 1	NM_000442.4	**PECAM1**	exon 5	exon 6	94
bone morphogenetic protein receptor type 2	NM_001204.6	**BMPR2**	exon 7	exon 8	98
snail family transcriptional repressor 1	NM_005985.3	**SNAI1**	Junction 1-2	exon 2	114
snail family transcriptional repressor 2	NM_003068.4	**SNAI2**	exon 3	exon 3	139
twist family bHLH transcription factor 1	NM_000474.3	**TWIST1**	exon 1	exon 1	86
interferon alpha 1	NM_024013.1	**IFNA1**	exon 1	exon 1	116
interleukin 18	NM_001562.3	**IL18**	Junction 5-6	exon 6	75
prostaglandin F receptor	NM_000959.4	**PTGFR**	exon 2	exon 2	125
angiotensin I converting enzyme	NM_000789.4	**ACE**	exon 14	exon 15	101
angiotensin converting enzyme 2	NM_021804.3	**ACE2**	exon 7	exon 8	136
endothelin 1	NM_001955.4	**EDN1**	exon 2	Junction 2-3	68
endothelin receptor type A	NM_001957.4	**EDNRA**	exon 2	exon 2	95
endothelin receptor type B	NM_000115.5	**EDNRB**	exon 2	exon 3	143
vascular endothelial growth factor A	NM_001025366.2	**VEGFA**	exon 3	exon 4	110
RNA, 18S ribosomal 5	NR_003286.2.1	**RNA18S5**	exon 1	exon 1	71

Supplemental Table 7: Primers used in RT-qPCR for hPASMCs.

Gene name	RefSeq	Symbol	Forw Primer location	Rev Primer location	Amplicon size (bp)
bone morphogenetic protein receptor, type II (serine/threonine kinase)	NM_001204.6	**BMPR2**	exon 7	exon 8	98
bone morphogenetic protein receptor, type IA	NM_004329.2	**BMPR1A**	exon 8	exon 9	149
phosphodiesterase 5A	NM_001083.4	**PDE5A**	exon 7	exon 8	129
transforming growth factor, beta receptor 1	NM_004612.2	**TGFBR1**	exon 4	exon 5	150
transforming growth factor, beta receptor II (70/80kDa)	NM_001024847.2	**TGFBR2**	exon 5	exon 6	97
transforming growth factor, beta receptor III	NM_003243.4	**TGFBR3**	exon 4	exon 5	112
endothelin receptor type A	NM_001957.4	**EDNRA**	exon 2	exon 2	95
endothelin receptor type B	NM_000115.5	**EDNRB**	exon 2	exon 3	143
RNA, 18S ribosomal 5	NR_003286.2.1	**RNA18S5**	exon 1	exon 1	71
actin, beta	NM_001101.3	**ACTB**	5' UTR	exon 2	101

Supplemental Table 8: Primers used for siRNA validation on hPASMCs and hPECs.

Primer	Reference	Supplier
Orai2 human	Hs00259863_m1	Thermo Fisher Scientific
Orai3 human	Hs00743683_s1	
18S human	Hs03003631_g1	

Supplemental Figures and Legends

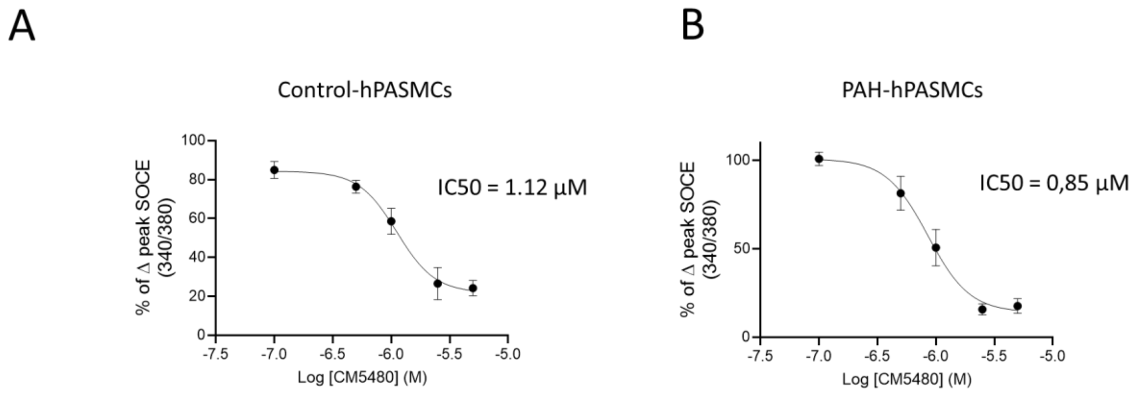

Supplemental Figure 1

Supplemental Figure 1: Effect of CM5480 on SOCE inhibition in control and PAH-hPASMCs. (**A**) Dose-response relationship of percentage of delta peak SOCE over the log of CM5480 concentration in M in control-hPASMCs (n=7 different patients). (**B**) Dose-response relationship of percentage of delta peak SOCE over the log of CM5480 concentration in M in PAH-hPASMCs (n=7 different patients).

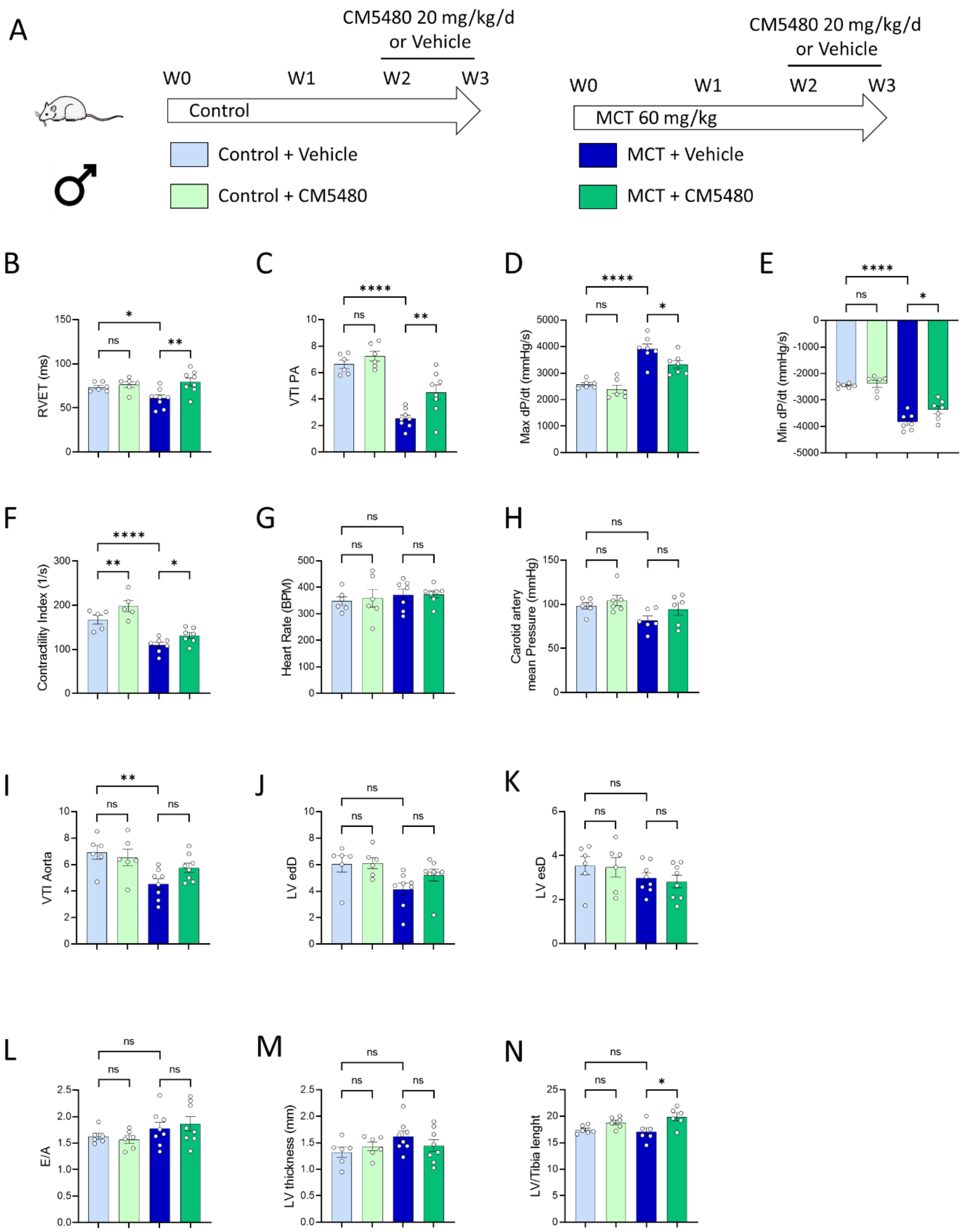

Supplemental Figure 2

Supplemental figure 2: Effect of inhibition of Orai1 with selective inhibitor CM5480 in male MCT-rats. (A) In vivo experimental design. Control and MCT male rats were treated with vehicle or CM5480 (20 mg/kg/day) by oral gavage for 7 days between week 2 and week 3. (**B**) RV ejection time (RVET) (n=6 for Control group, n=8 for MCT group), (**C**) VTI PA n=6

for Control group, n=8 for MCT group), (**D**) max dP/dt in mmHg/s n=6 for Control group, n=7 for MCT group), (**E**) min dP/dt in mmHg/s n=6 for Control group, n=8 for MCT group), (**F**) contractility index in 1/s n=5 for Control group, n=7 for MCT group), (**G**) heart beat in BPM (beats per minute) n=6 for Control group, n=7 for MCT group), (**H**) carotid artery mean pressure in mmHg (n=6 for all groups), (**I**) velocity time integral (VTI) aorta (n=6 for all groups), (**J**) LV edD n=6 for Control group, n=8 for MCT group), (**K**) LVesD n=6 for Control group, n=8 for MCT group), (**L**) E/A ratio n=6 for Control group, n=8 for MCT group), (**M**) LV thickness (n=6 for Control group, n=8 for MCT group), (**N**) LV/tibia length (n=6 for all groups). Mean + SEM. One-way ANOVA, Tukey post-hoc. ns = non significant; * *P*<0.05, ***P*<0.01, ***P<0.001, **** *P*<0.0001.

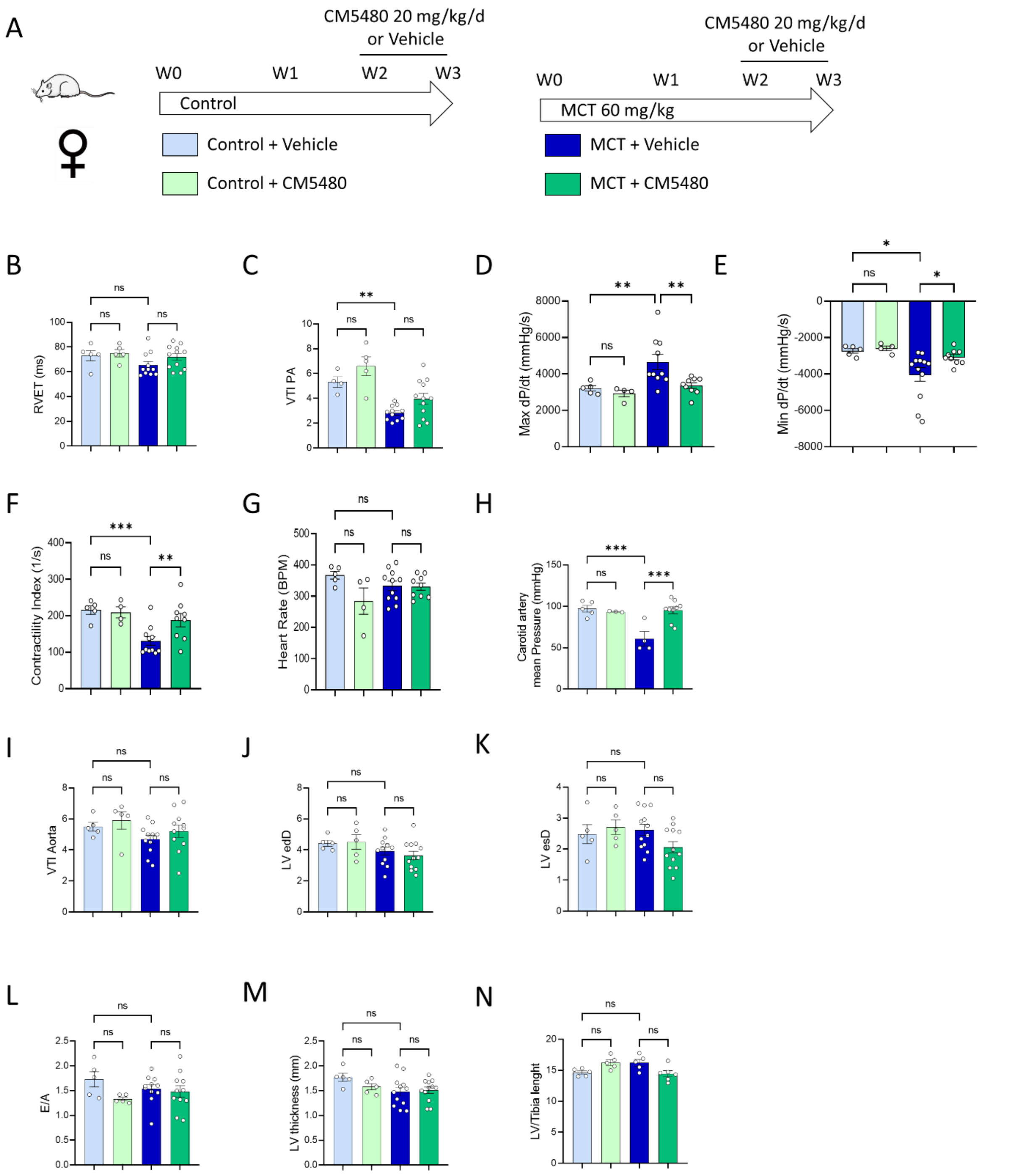

Supplemental Figure 3

Supplemental figure 3: Effect of inhibition of Orai1 with selective inhibitor CM5480 in female MCT-rats. (A) In vivo experimental design. Control and MCT female rats were treated with vehicle or CM5480 (20mg/kg/day) by oral gavage for 7 days between week 2 and week 3. (**B**) RVET in ms (n=5 for Control+Vehicle and Control+CM5480, n=11 for MCT+Vehicle and n=12 for MCT+CM5480), (**C**) VTI PA (n=4 for Control+Vehicle, n=5 Control+CM5480, n=11

for MCT+Vehicle and n=12 for MCT+CM5480), (**D**) max dP/dt in mmHg/s (n=5 for Control+Vehicle, n=4 control+CM5480, n=10 for MCT+Vehicle, n=9 for MCT+CM5480), (**E**) min dP/dt in mmHg/s (n=5 for Control+Vehicle, n=4 Control+CM5480, n=12 for MCT+Vehicle, n=9 for MCT+CM5480), (**F**) contractility index in 1/s (n=5 for Control+Vehicle, n=4 Control+CM5480, n=11 for MCT+Vehicle, n=9 for MCT+CM5480), (**G**) heart beat in BPM (n=5 for Control+Vehicle, n=4 Control+CM5480, n=11 for MCT+Vehicle, n=9 for MCT+CM5480), (**H**) carotid artery mean pressure in mmHg (n=5 for Control+Vehicle, n=3 control+CM5480, n=4 for MCT+Vehicle, n=9 for MCT+CM5480), (**I**) VTI aorta (n=5 for Control+Vehicle and Control+CM5480, n=11 for MCT+Vehicle and n=12 for MCT+CM5480), (**J**) LV edD (n=5 for Control+Vehicle and Control+CM5480, n=12 for MCT+Vehicle, n=11 for MCT+CM5480), (**K**) LV esD (n=5 for Control+Vehicle and Control+CM5480, n=12 for MCT+Vehicle and MCT+CM5480), (**L**) E/A ratio (n=5 for Control+Vehicle and Control+CM5480, n=11 for MCT+Vehicle, n=12 for MCT+CM5480), (**M**) LV thickness in mm (n=5 for Control+Vehicle and Control+CM5480, n=12 for MCT+Vehicle and MCT+CM5480). (**N**) LV/tibia length (n=6 for all groups).). Mean + SEM. One-way ANOVA, Tukey post-hoc. ns = non significant; * *P*<0.05, ***P*<0.01, ***P<0.001.

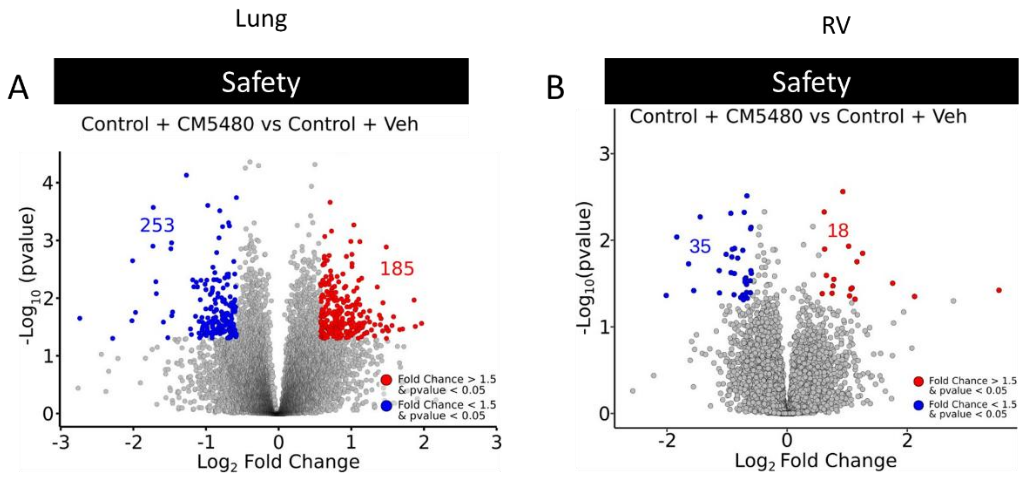

Supplemental Figure 4

Supplemental figure 4: Molecular consequence of CM5480 treatment on lungs and RV on control rats. (**A**) Volcano plot showing gene differentially expressed in the lungs from Control+CM5480 vs. Control+Vehicle male rats expressed as -Log10(p-value) over Log2FC. In red, 185 upregulated genes with a fold change (FC) > 1.5 and p-value < 0.05. In blue, 253 genes downregulated with a FC < 1.5 and p-value < 0.05. (**B**) Volcano plot showing gene differentially expressed in RV from Control+CM5480 vs. Control+Vehicle male rats as -Log10(p-value) over Log2FC. In red, 18 genes upregulated with a FC > 1.5 and p-value < 0.05. In blue, 35 genes down-regulated with a FC < 1.5 and p-value < 0.05. Statistical hypothesis testing used Deseq2 R package with Wald test and the Benjamini-Hochberg method for multiple testing.

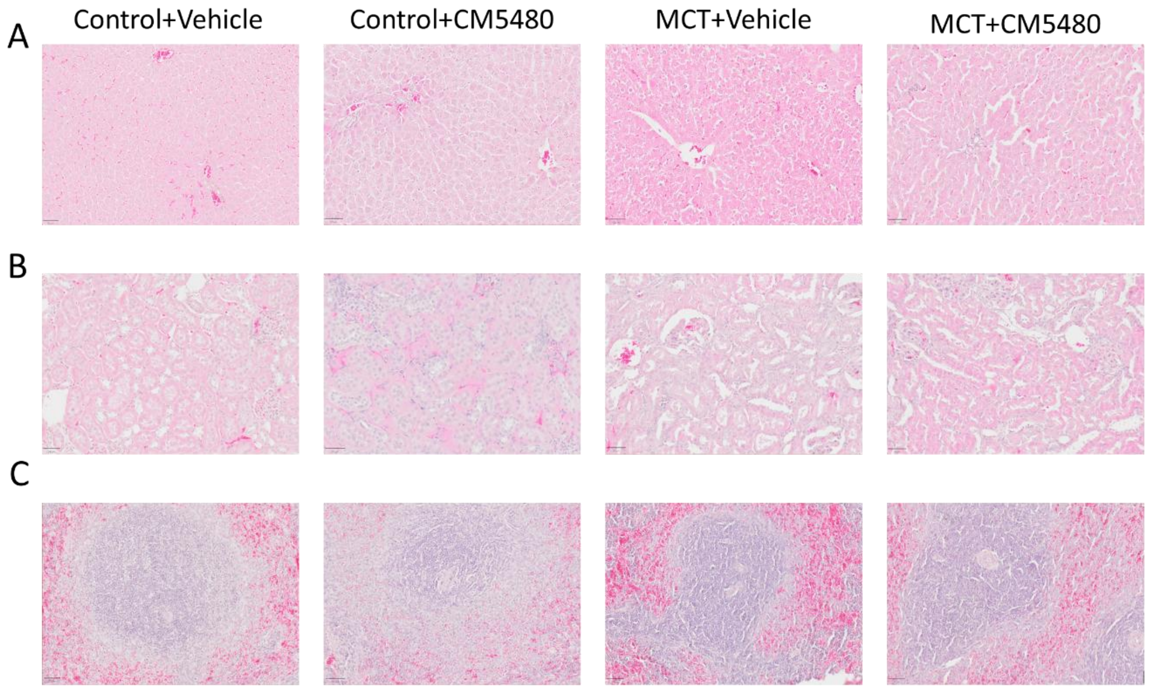

Supplemental Figure 5

Supplemental figure 5: Consequence of inhibition of Orai1 with selective inhibitor CM5480 in kidney, spleen, and liver in male Control+Vehicle, Control+CM5480, MCT+Vehicle and MCT+CM5480 experimental groups. Hematoxylin/eosin/Safran

staining of paraffin-embedded liver (**A**) kidney (**B**), and spleen (**C**) sections from the Control+Vehicle, Control+CM5480, MCT+Vehicle and MCT+CM5480 groups.

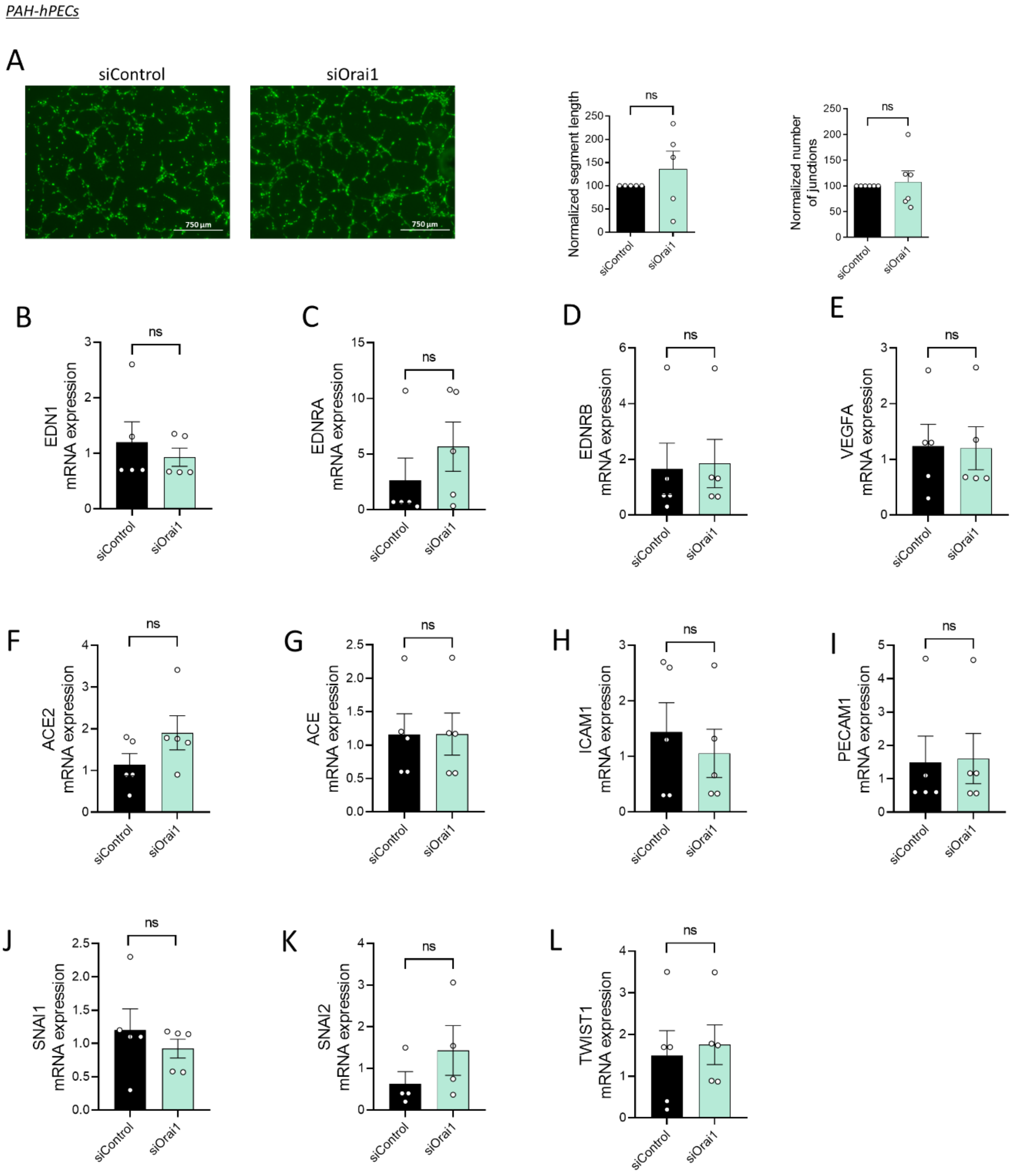

Supplemental Figure 6

Supplemental figure 6: Consequence of Orai1 knockdown in PAH-hPECs. (**A**) Representative images of endothelial tube formation assay in PAH-hPECs transfected with siControl or siOrai1. Bar graph = 750 µm. Quantification of normalized segment length and number of junctions (n=6 patients). mRNA expression of (**B**) EDN1, (**C**) EDNRA, (**D**)

EDNRB, (**E**) VEGFA, (**F**) ACE2 (**G**) ACE, (**H**) ICAM1, (**I**) PECAM1 (**J**) SNAI1 (**K**) SNAI2 and (**L**) TWIST1 in PAH-hPECs transfected with siControl or siOrai1 (n=5 patients for all graphs). Mean + SEM. T-test or Mann-Whitney test. ns = non significant.

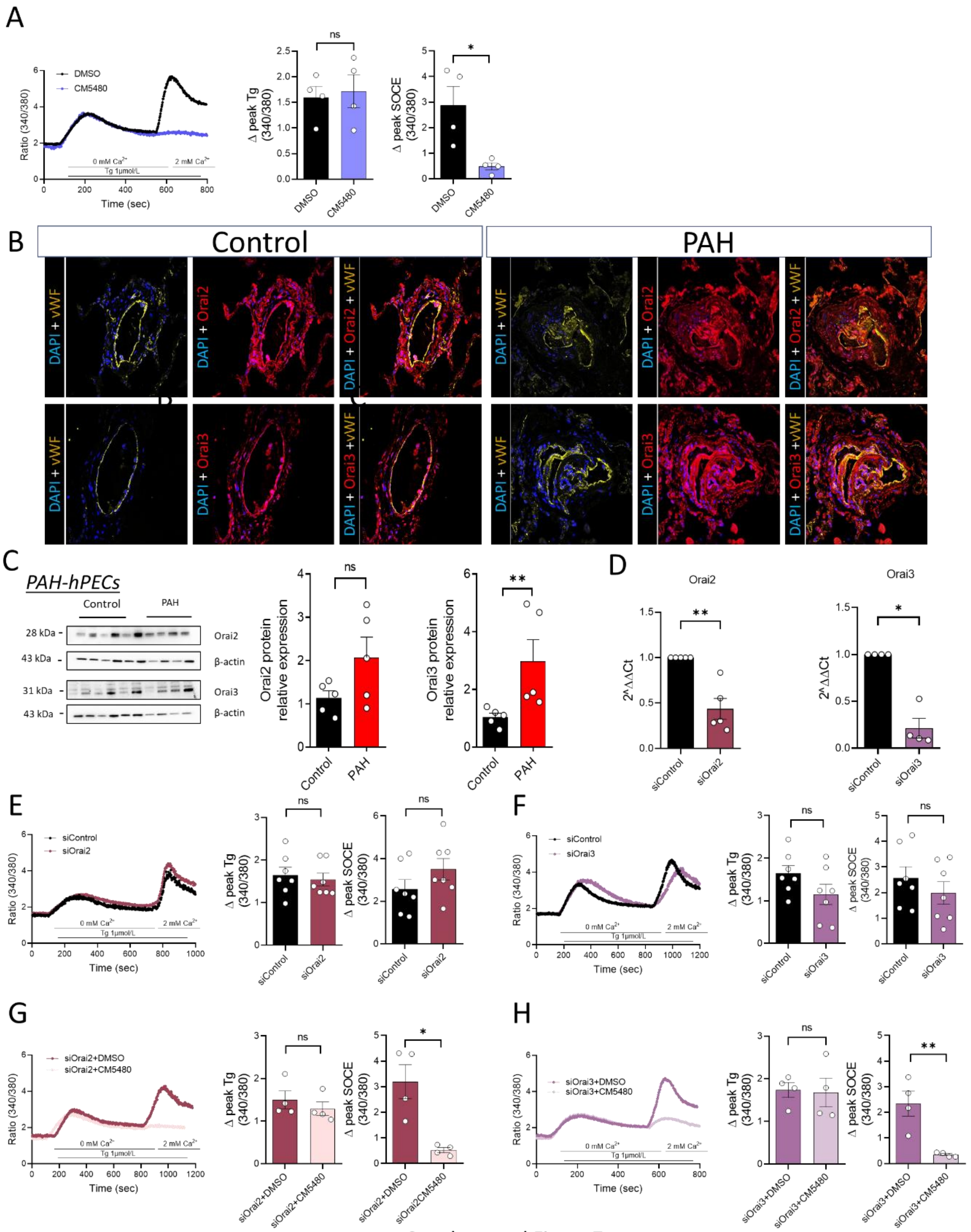

Supplemental Figure 7

Supplemental figure 7: Role of Orai2 and Orai3 in PAH-hPECs. (**A**) Consequences of Orai1 inhibition with CM5480 (1 µmol/L) in PAH-hPECs on ER Ca^{2+} release (n=4 patients) and on SOCE amplitude triggered by Thapsigargin (Tg) (n=4 patients). (**B**) Localization of Orai2 and Orai3 expression by immunofluorescence staining of paraffin-embedded lung sections from control and PAH patients. Orai2 or Orai3 are green, vWF is yellow to localize hPECs, and nuclei are blue (DAPI). (**C**) Immunoblot images and quantification of Orai2 and Orai3 protein expression in control and PAH-hPECs (n=5 patients for each condition). (**D**) Validation of siRNA efficency in PAH-hPECs transfected with siControl or siOrai2 or siOrai3 on mRNA expression of Orai2 and Orai3 respectively (n=5). (**E**) Consequences of Orai2 knockdown in PAH-hPECs on ER Ca^{2+} release (n=7 patients) and on SOCE amplitude triggered by Tg (n=7 patients). (**F**) Consequences of Orai3 knockdown in PAH-hPECs on ER Ca^{2+} release (n=7 patients) and on SOCE amplitude triggered by Tg (n=7 patients). (**G**) Consequences of Orai1 inhibition with CM5480 (1 µmol/L) on Orai2 knockdown in PAH-hPECs on ER Ca^{2+} release (n=4 patients) and on SOCE amplitude triggered by Tg (n=4 patients). (**H**) Consequences of Orai1 inhibition with CM5480 (1 µmol/L) on Orai3 knockdown in PAH-hPECs on ER Ca^{2+} release (n=4 patients) and on SOCE amplitude triggered by Tg (n=4 patients). Mean + SEM. T-test or Mann-Whitney test. ns = non significant; * $P<0.05$, ** $P<0.01$.

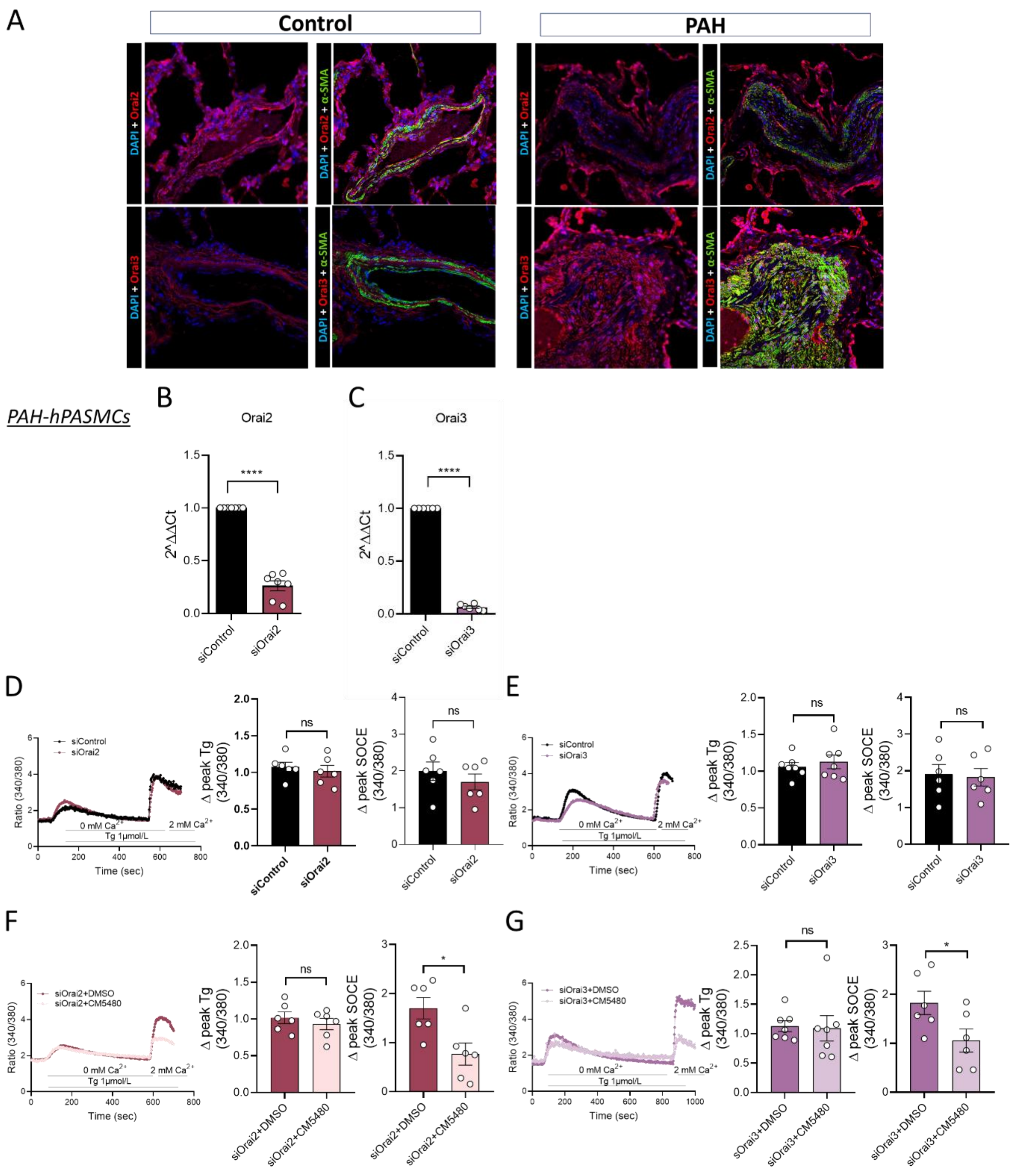

Supplemental Figure 8

Supplemental figure 8: Role of Orai2 and Orai3 in PAH-hPASMCs. (**A**) Localization of Orai2 and Orai3 expression by immunofluorescence staining of paraffin-embedded lung sections from control and PAH patients. Orai2 or Orai3 are green, α-SMA is green to localize hPASMCs, and nuclei are blue (DAPI). (**B**) Validation of siRNA efficency in PAH-hPASMCs transfected with siControl or siOrai2 on mRNA expression of Orai2 (n=7). (**C**) Validation of

siRNA efficency in PAH-hPASMCs transfected with siControl or siOrai3 on mRNA expression of Orai3 (n=6). (**D**) Consequences of Orai2 knockdown in PAH-hPASMCs on ER Ca^{2+} release (n=6 patients) and on SOCE amplitude triggered by Thapsigargin (Tg) (n=6 patients). (**E**) Consequences of Orai3 knockdown in PAH-hPASMCs on ER Ca^{2+} release (n=7 patients) and on SOCE amplitude triggered by Tg (n=6 patients). (**F**) Consequences of Orai1 inhibition with CM5480 (1 µmol/L) on Orai2 knockdown in PAH-hPASMCs on ER Ca^{2+} release (n=6 patients) and on SOCE amplitude triggered by Tg (n=6 patients). (**G**) Consequences of Orai1 inhibition with CM5480 (1 µmol/L) on Orai3 knockdown in PAH-hPASMCs on ER Ca^{2+} release (n=7 patients) and on SOCE amplitude triggered by Tg (n=6 patients). Mean + SEM. T-test or Mann-Whitney test. ns = non significant; * *P*<0.05, ***P*<0.01, ***P<0.001.

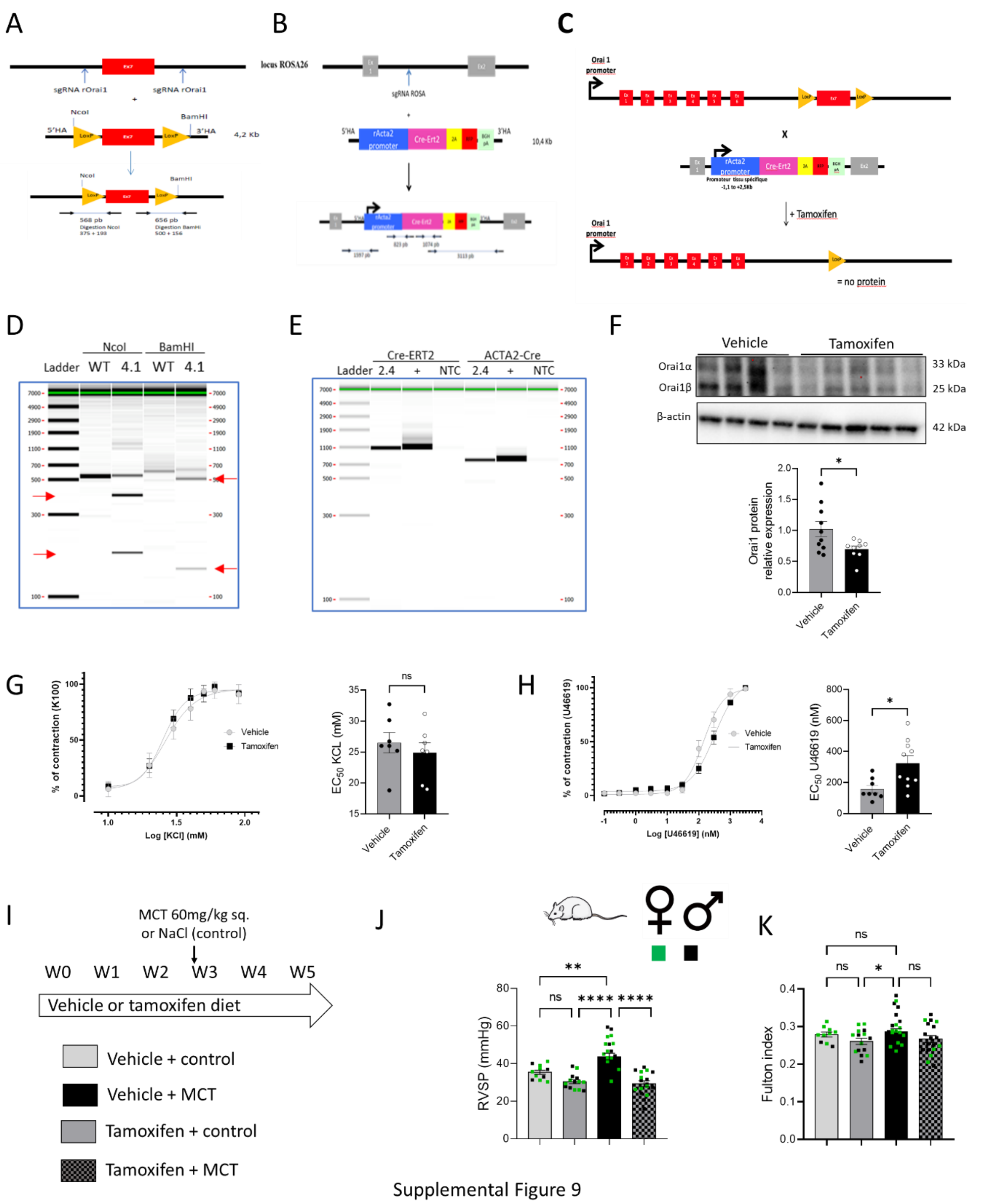

Supplemental Figure 9

Supplemental figure 9: SMC-Orai1 deficient rats are protected against MCT-induced PAH. (**A**) Schematic illustration of the genetic design of line rOrai1_LoxP knock-in rats. (**B**) Illustration of the genetic design of line rActa2-CreERT2RFP/ROSA knock-in rats. (**C**) Strategy use to generate rOrai1 knock-in rats after tamoxifen induction. (**D-E**) Genotypes for all founder rats (related to Fig. 1A et 1B). (**D**) PCR genotyping of rat line rOrai1_LoxP knock-

in rats were resolved by electrophoresis. PCR products were digested by NcoI (WT = 533 bp; Knock-in (KI) = 375 + 193 bp) or by BamHI (WT = 620 bp; KI = 500 + 156 bp) corresponded with the expected band sizes. (**E**) PCR Genotyping of rActa2-CreERT2/ROSA knock-in rats were resolved by electrophoresis. PCR-specific products for the Cre-ERT2 (1074 pb) and for the ACTA2-Cre (823 pb). NTC: No Template Control (**F**) Immunoblot images and quantification of Orai1 protein expression in aorta of Control+Vehicle (n=10) and Control+tamoxifen treated rats (n=9). (**G, left**) Dose-response curve (normalized to K100) was established by applying increasing concentrations of KCl to isolated rat PAs from Control+Vehicle and Control+tamoxifen rats. (**G, right**) Corresponding quantification of EC_{50} (n=7 rats for Control+Vehicle and Control+tamoxifen). (**H, left**) Dose-response curve (normalized to U46619) was established by applying increasing concentrations of U46619 to isolated rat PAs from Control+Vehicle (n=8) and Control+tamoxifen rats (n=10). (**H, right**) Corresponding quantification of EC_{50}. (**I**) In vivo experimental design. Rats were feed with vehicle diet or tamoxifen diet for 5 weeks and PAH was induced by MCT injection (60 mg/kg sq.) or NaCl was injected for control rats on day 21. Males rats are represented in blacks quares and females in green squares. (**J**) RVSP, (**K**) Fulton index. Mean +SEM. T-test or Mann-Whitney test (**F**, **G** and **H**). One-way ANOVA (**J** and **K**). ns = non significant; * $P<0.05$, ** $P<0.01$, ***P<0.001, **** $P<0.0001$.

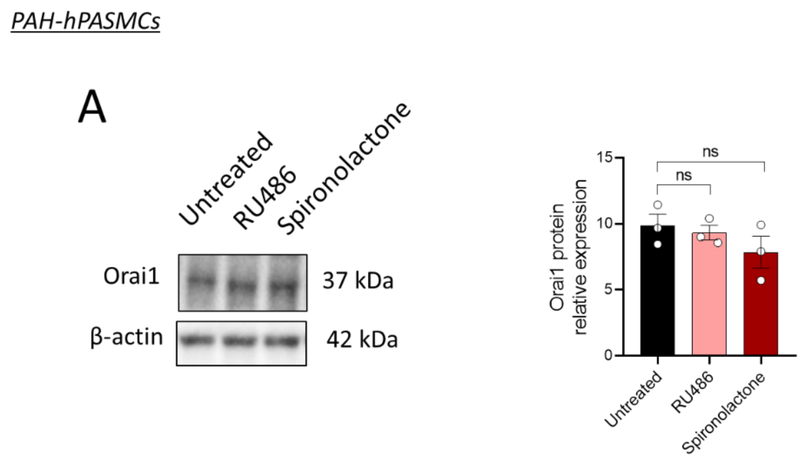

Supplemental Figure 10

Supplemental figure 10: Effect of RU486 and spironolactone treatment on Orai1 protein expression in PAH-hPASMCs. (**A**) Immunoblot images and quantification of Orai1 protein expression in PAH-hPASMCs treated with RU486 (10 µmol/L) or spironolactone (10 µmol/L) (n=3). Mean + SEM. One-way ANOVA. ns = non significant.
