# Supplementary material for: Combination of Orai1 inhibitor CM5480 with specific therapy mitigates pulmonary hypertension and its cardiac dysfunction
Source: JCI Insight. 2025 Nov 10;10(21):e191780. doi: 10.1172/jci.insight.191780 (PMC12643499; doi:10.1172/jci.insight.191780)

Full unedited blot for Figure 6C :

Anti-PDE5 1/1000

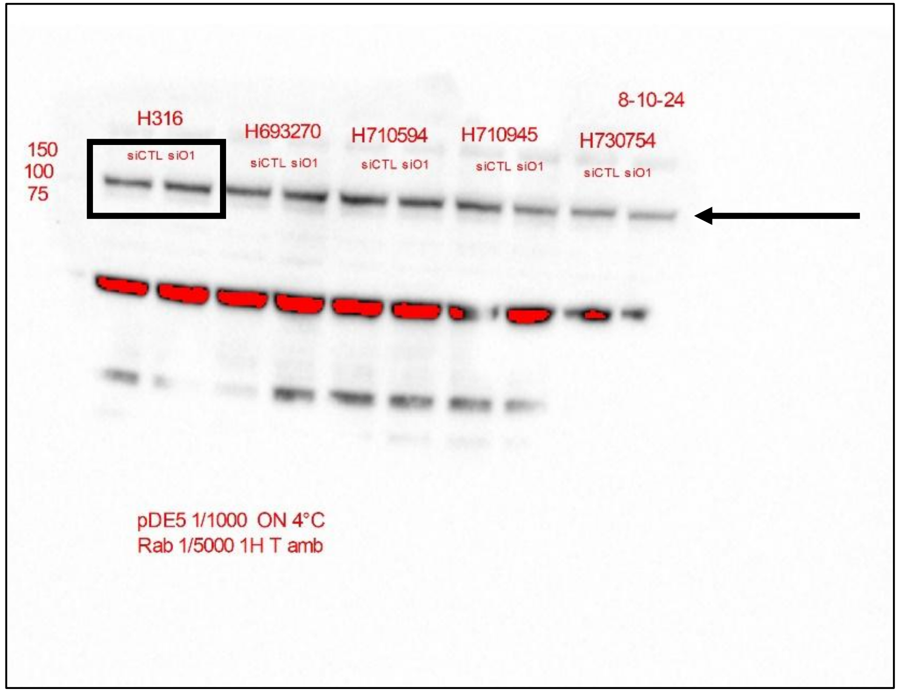

Anti-βactin 1/3000

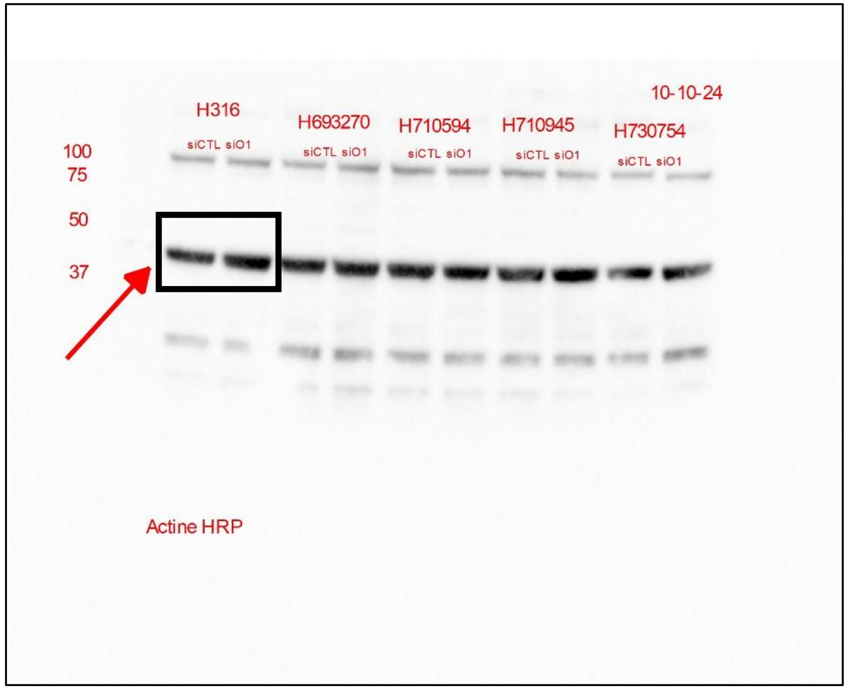

Full unedited blot for Figure 6F :

Anti-pSMAD1/5/9 1/1000

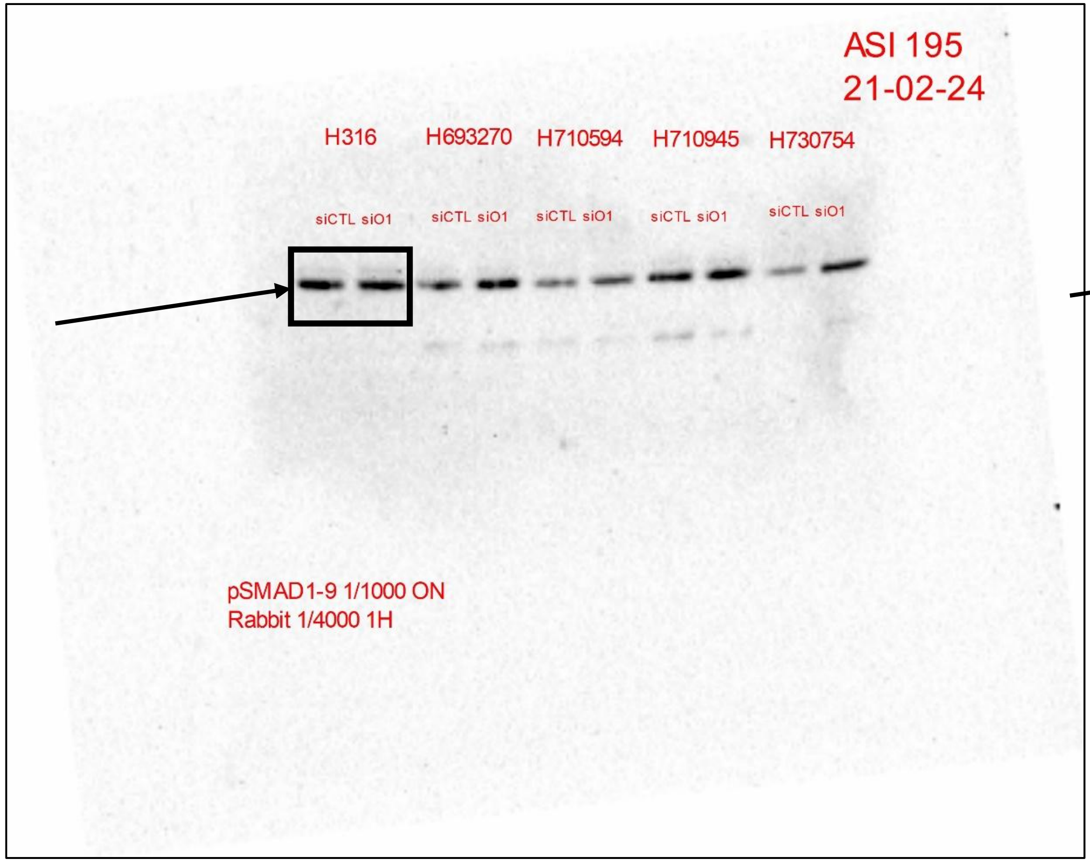

Anti-SMAD1 1/1000

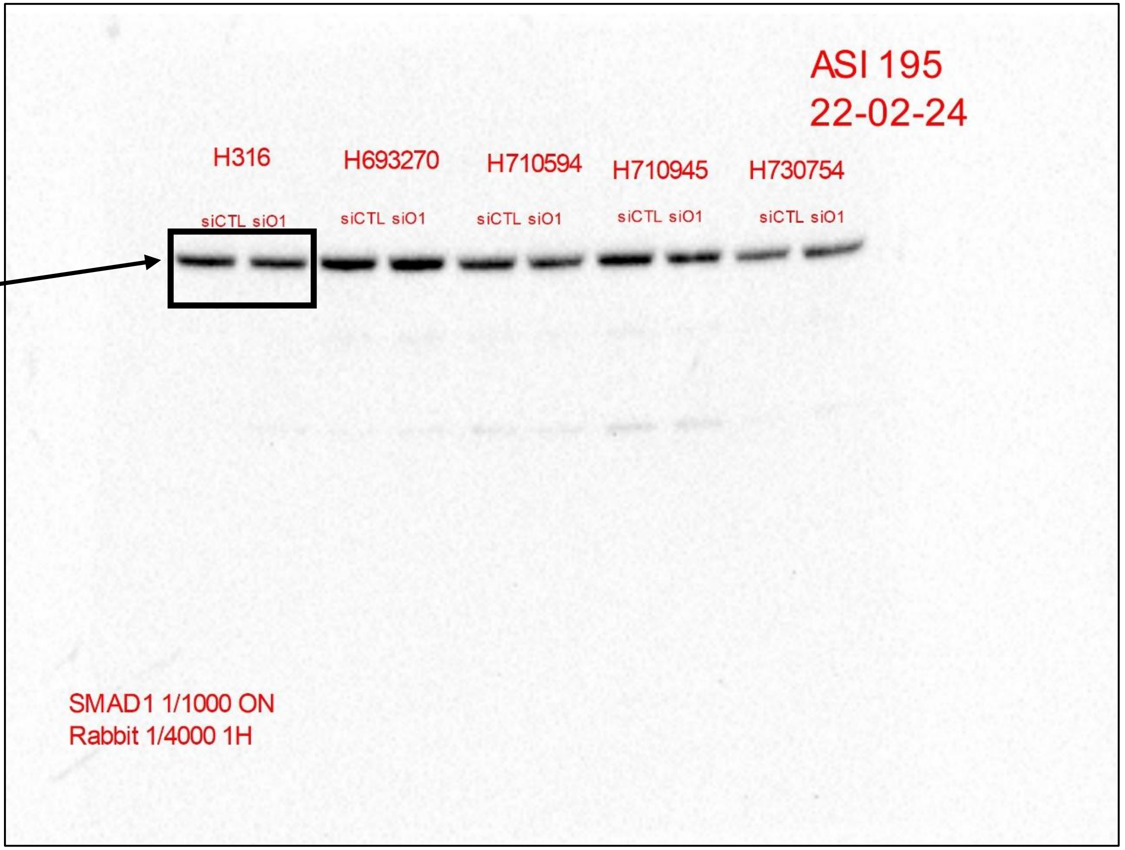

Full unedited blot for Figure 6G :

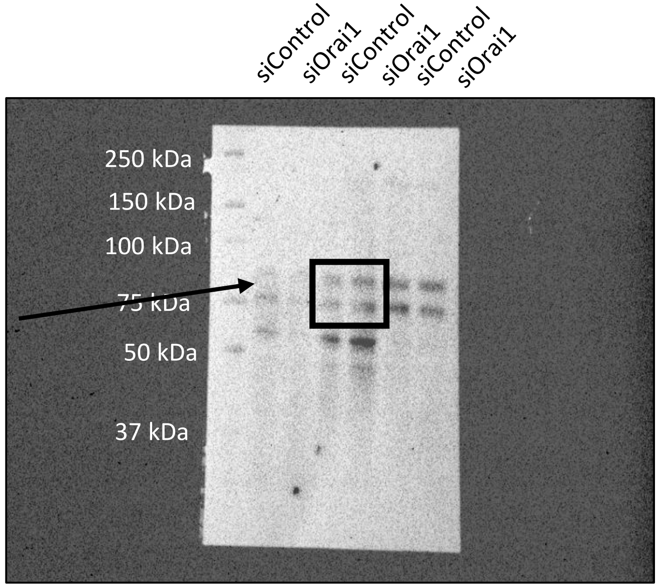

Anti-pSMAD2/3 1/1000

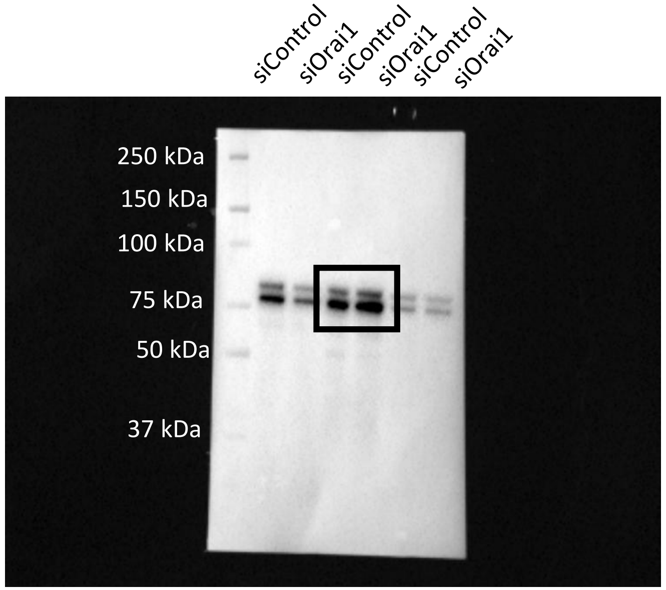

Anti-SMAD2/3 1/1000

Full unedited blot for Figure 6H :

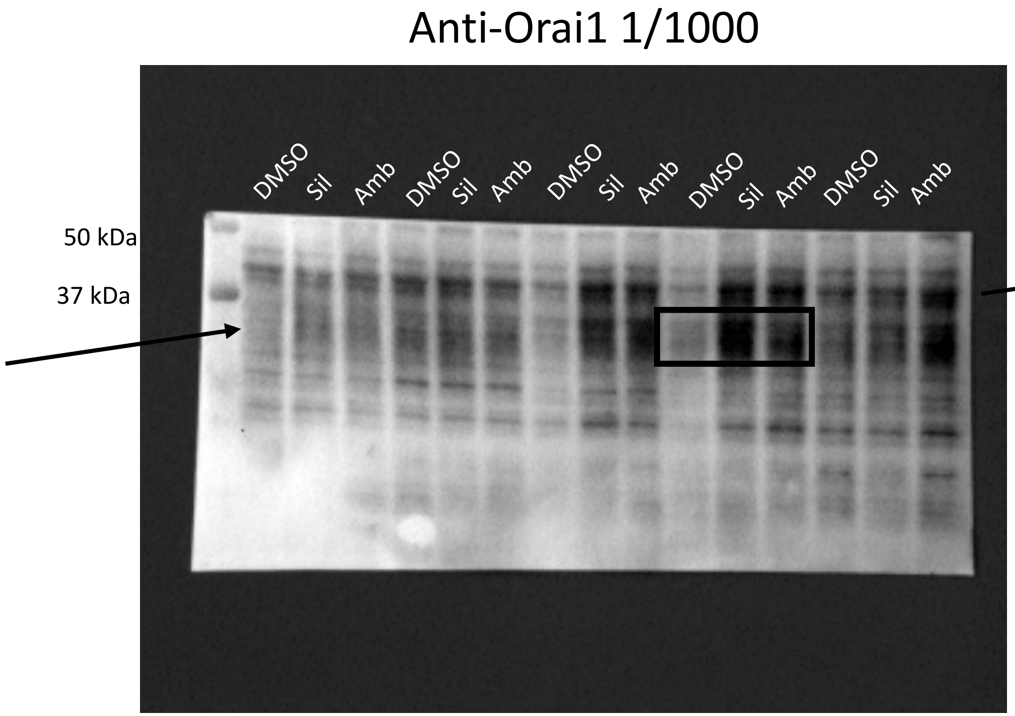

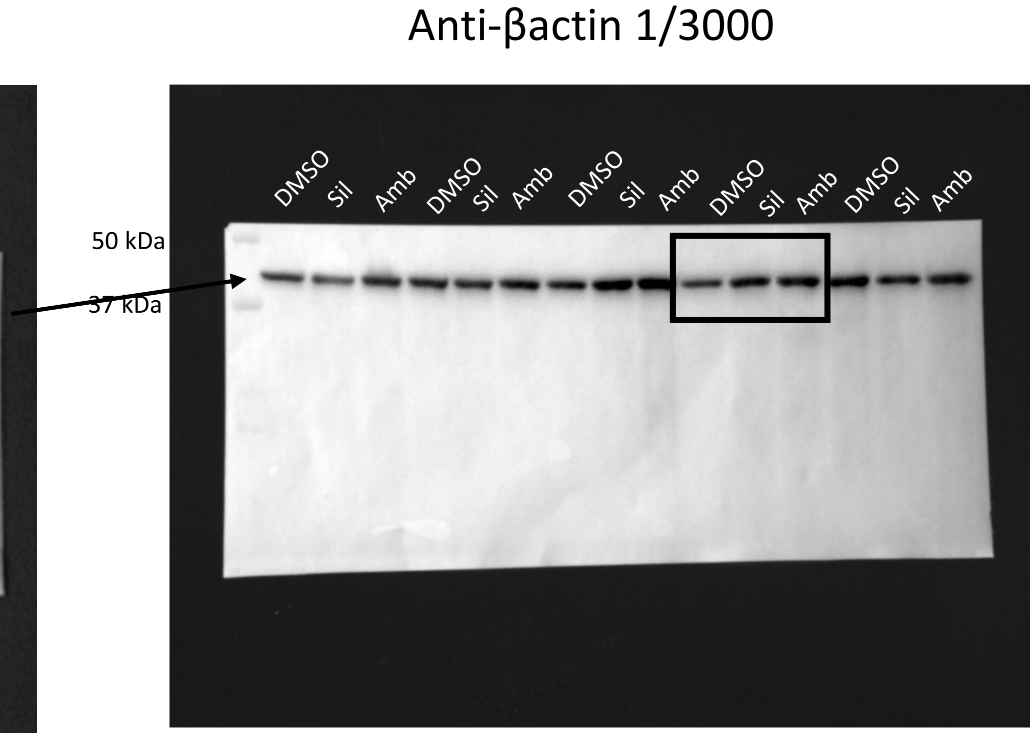

Full unedited blot for Figure 6I :

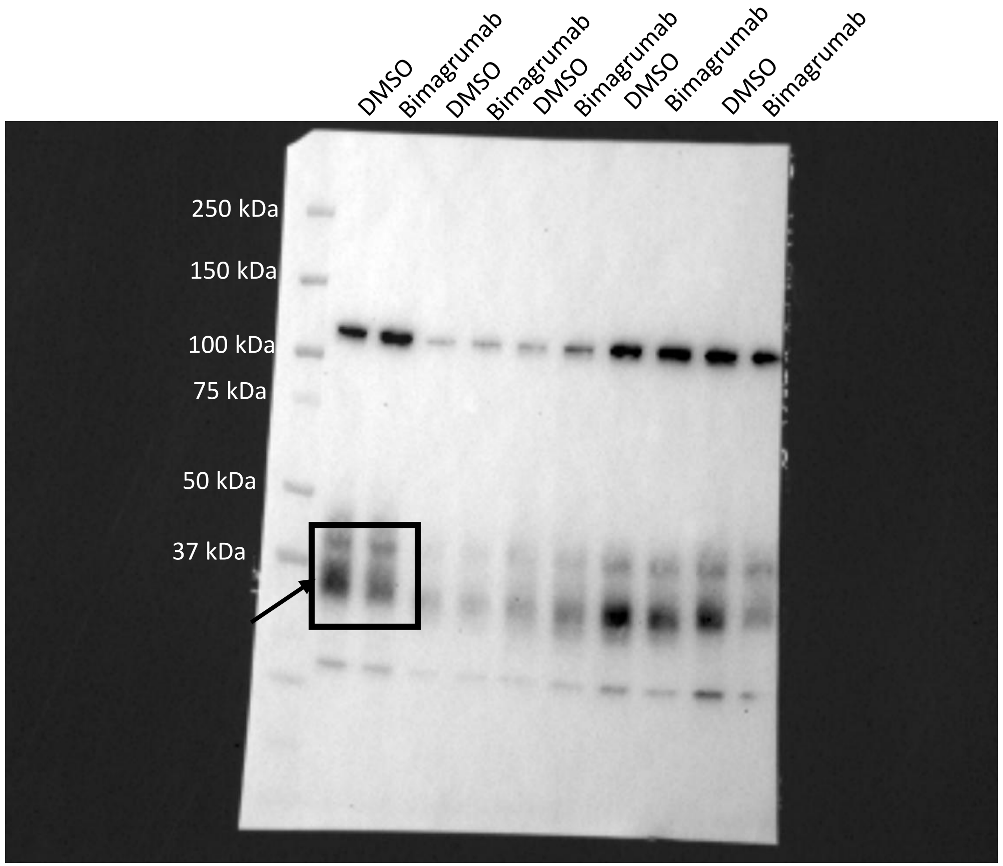

Anti-Orai1 1/1000

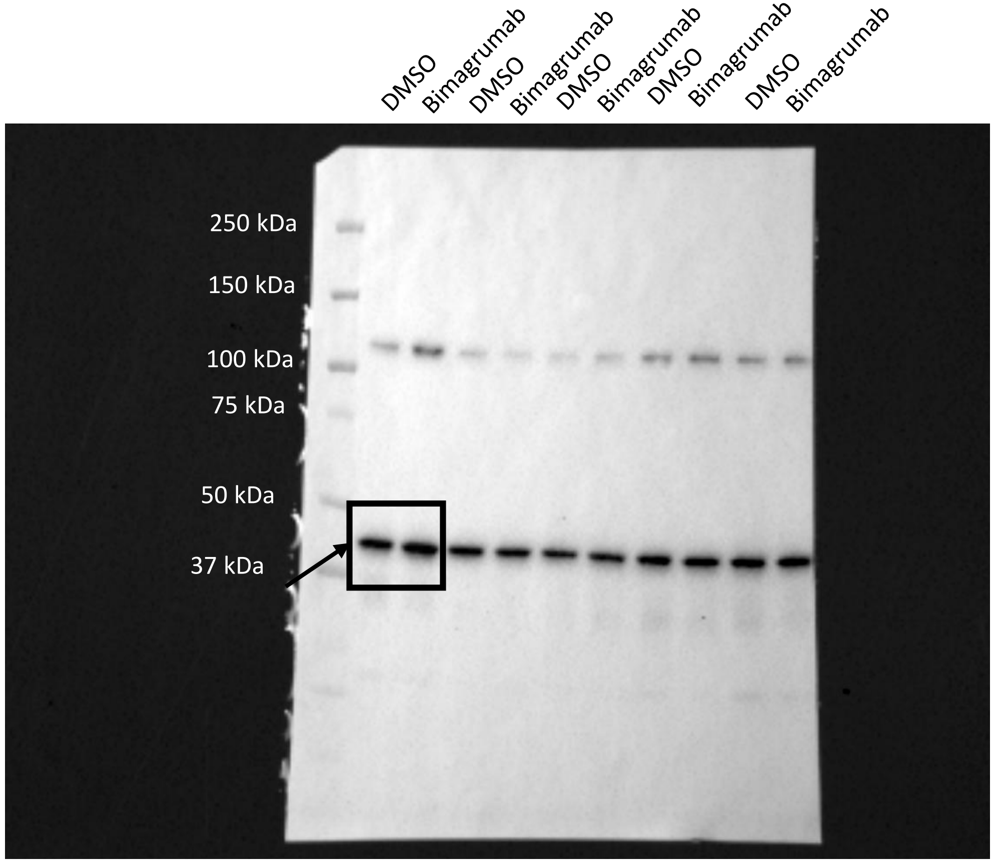

Anti-βactin 1/3000

Full unedited blot for Figure 6J : DMSO/Imatinib Orai1

Anti-Orai1 1/1000

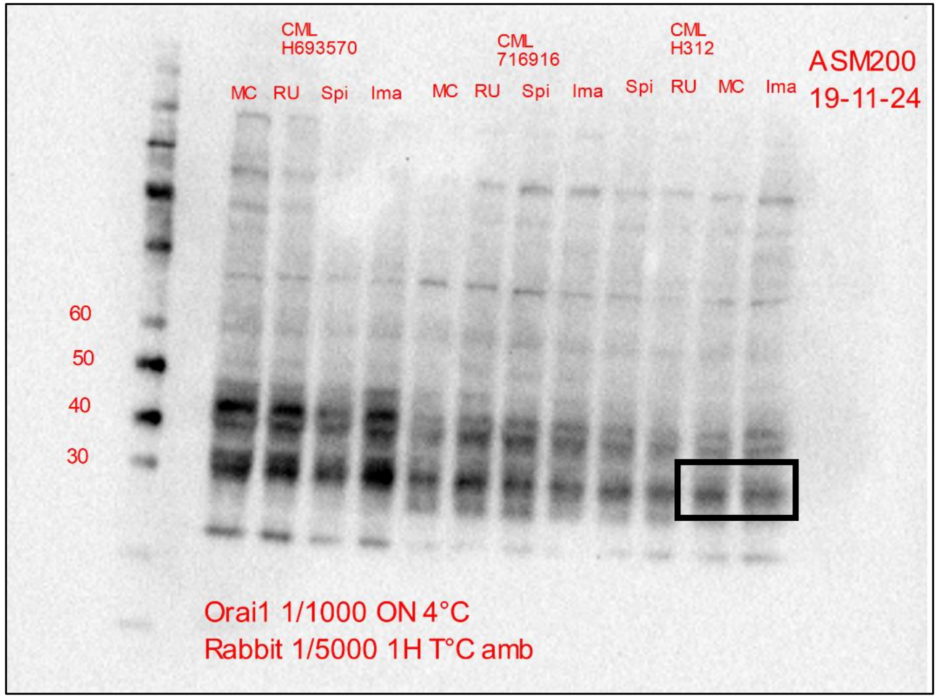

Anti-βactin 1/3000

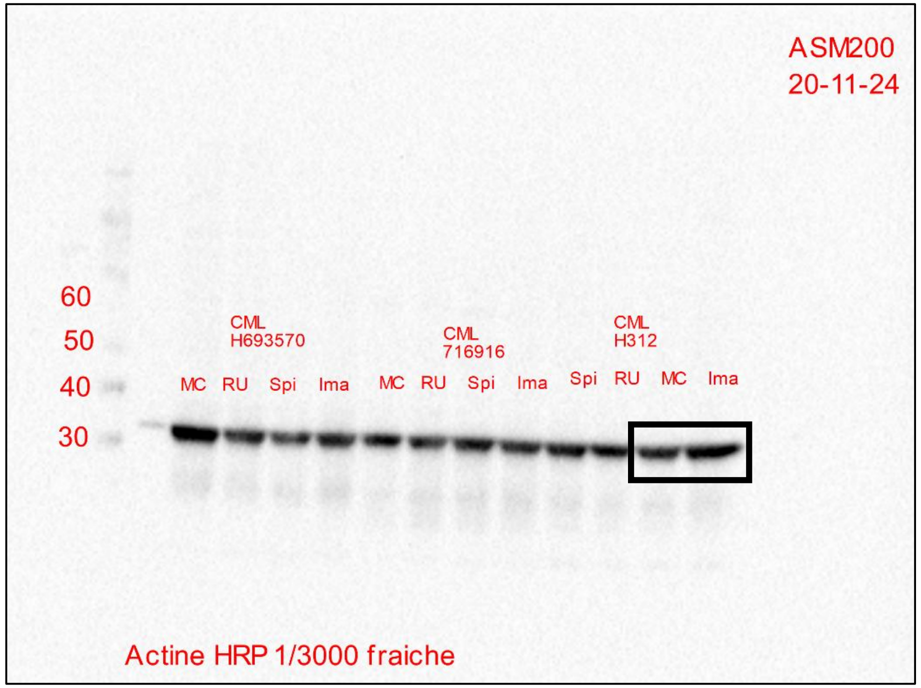

Full unedited blot for Figure 8A :

Anti-Orai1 1/1000

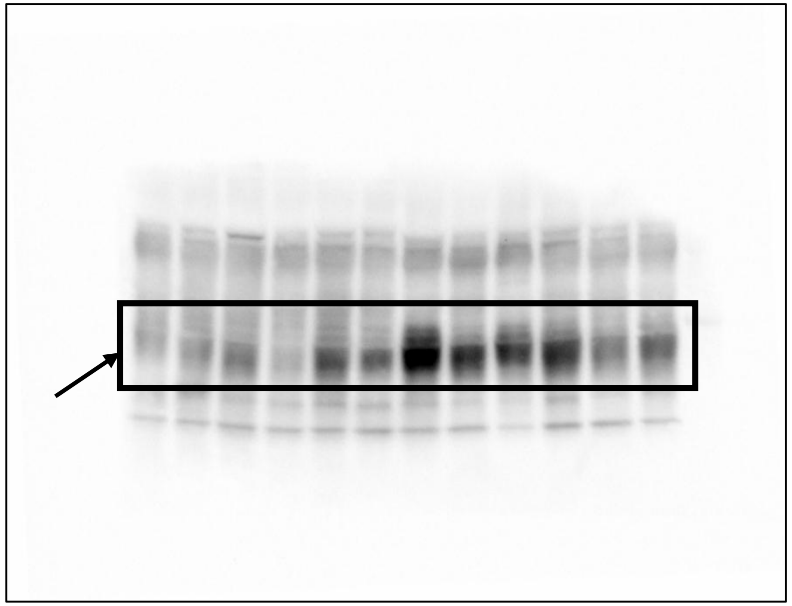

Anti-βactin 1/3000

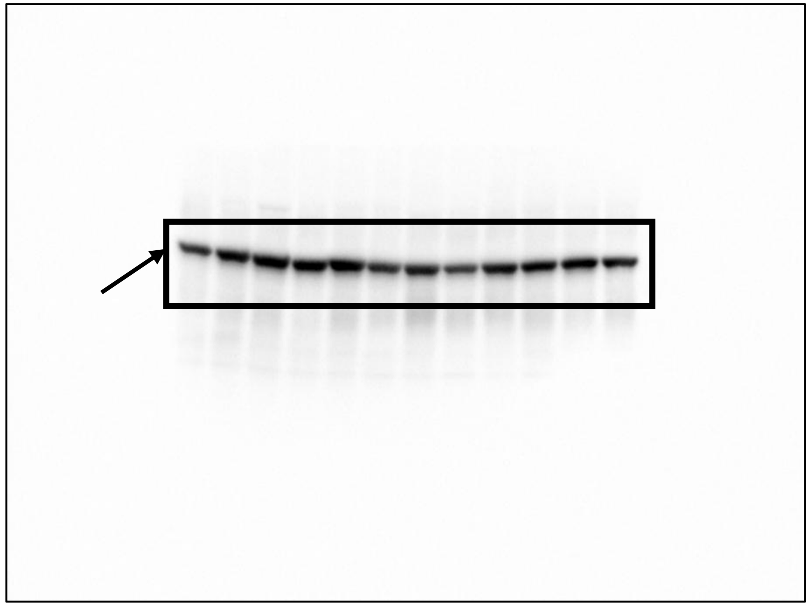

Full unedited blot for Supplemental Figure 7A :

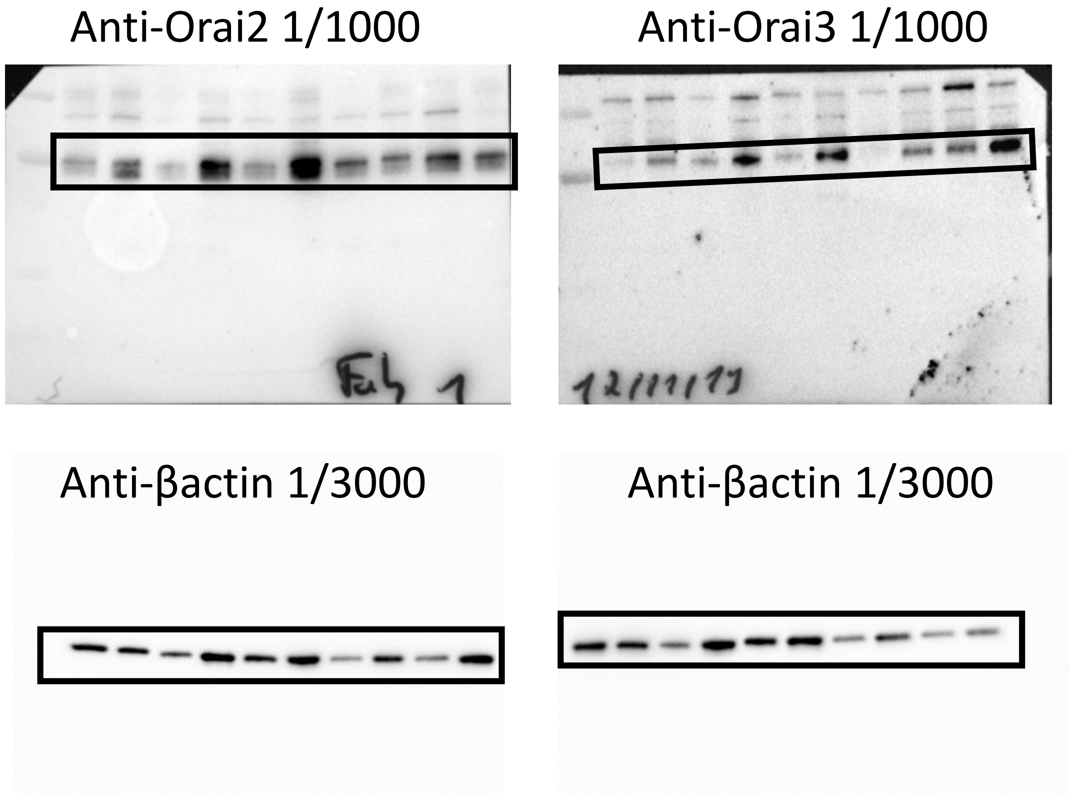

Full unedited blot for Supplemental Figure 9A : Orai1

Vehicle Tamoxifen

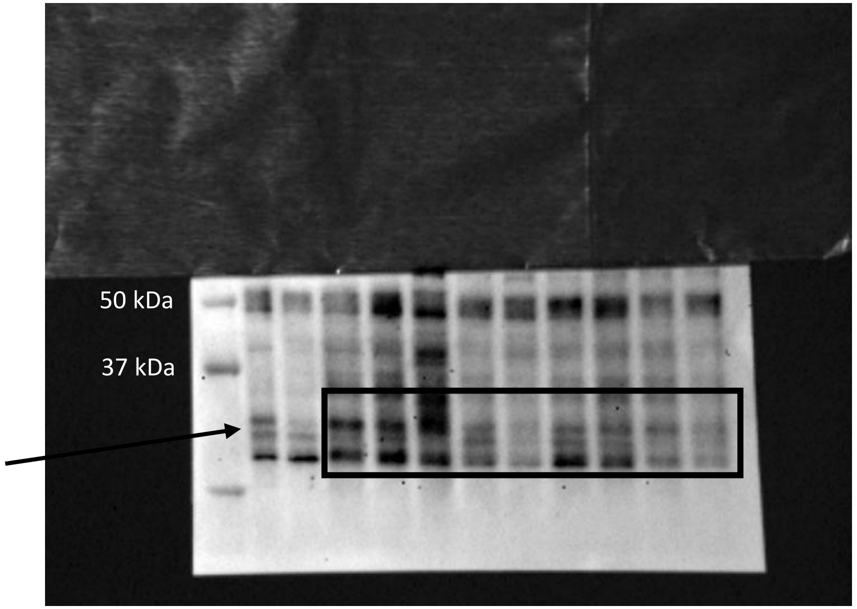

Anti-Orai1 1/1000

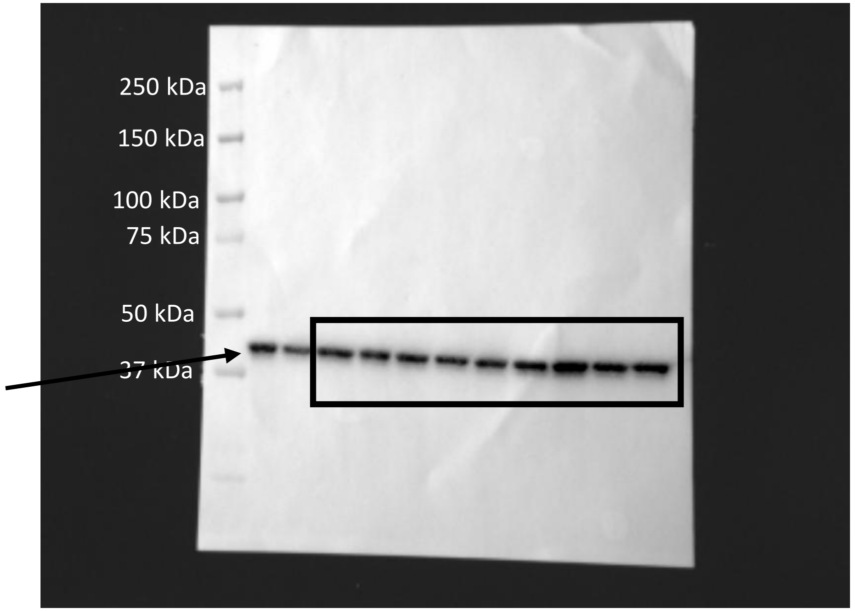

Anti-βactin 1/3000

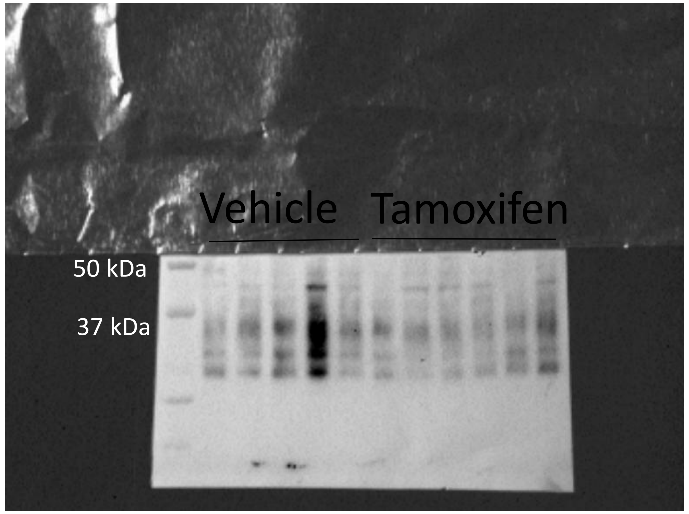

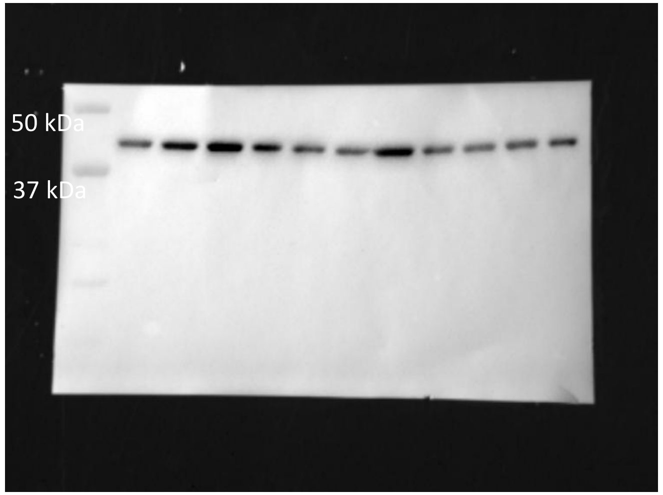

Full unedited blot for Supplemental Figure 10 : Orai1

Anti-Orai1 1/1000

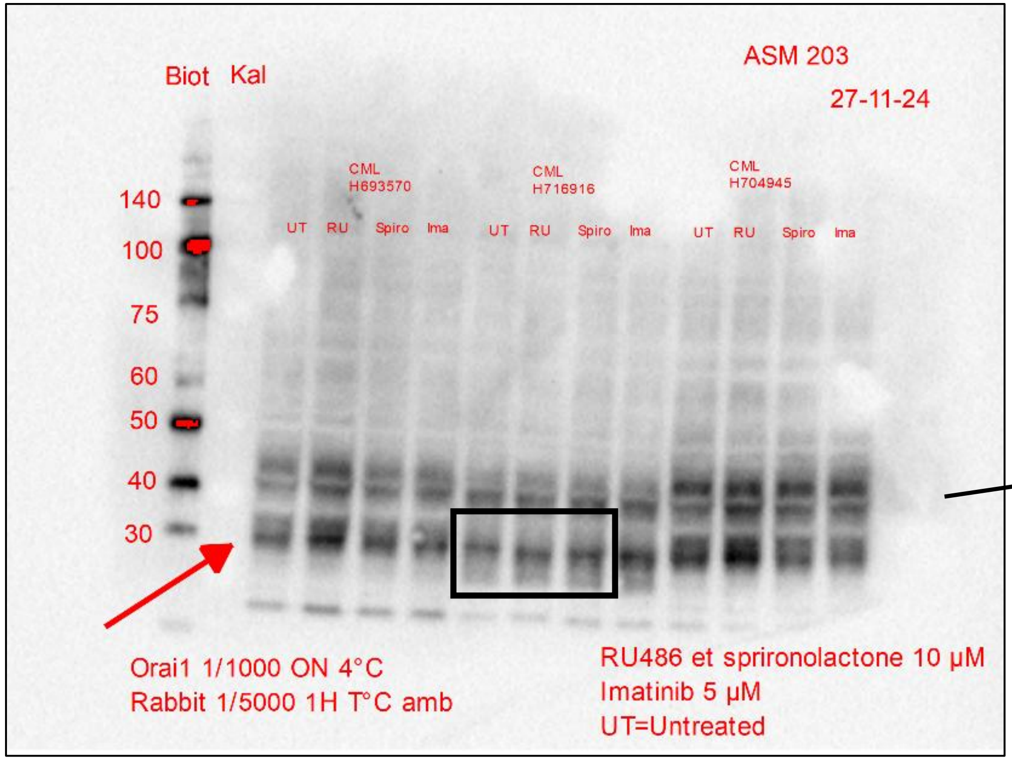

Anti-βactin 1/3000

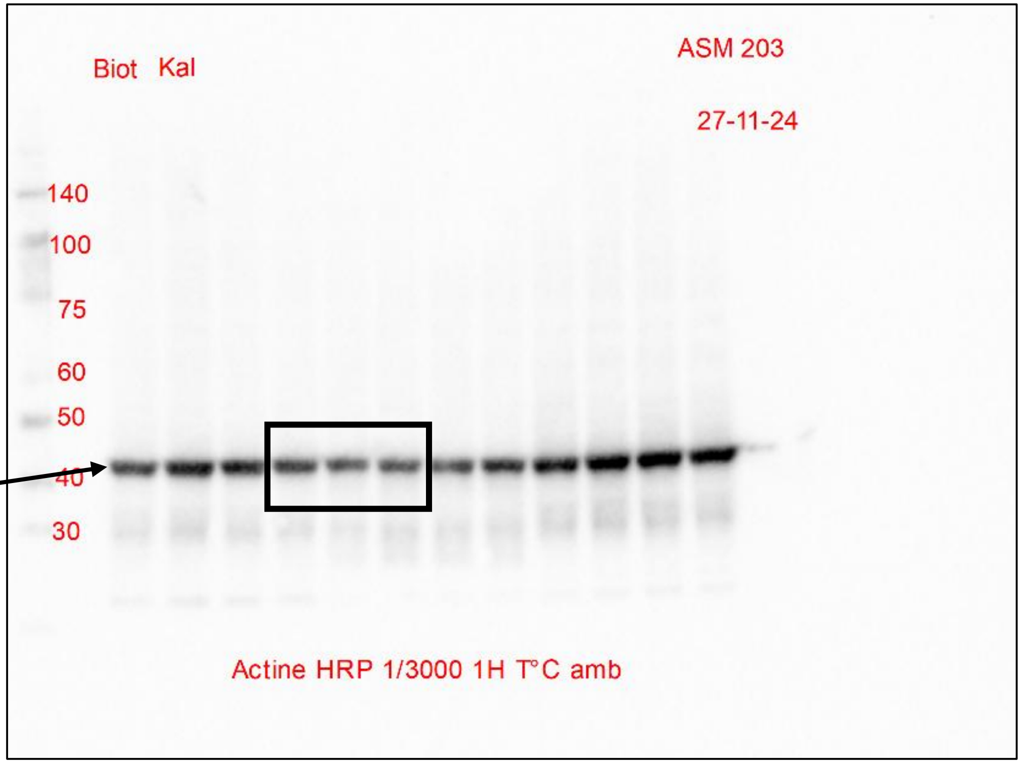

Supplement: Unedited blot and gel images [file jciinsight-10-191780-s174.pdf]
